# Supplementary figures and images for: Gas6 ameliorates intestinal mucosal immunosenescence to prevent the translocation of a gut pathobiont, Klebsiella pneumoniae, to the liver
Source: PLoS Pathog. 2023 Jun 8;19(6):e1011139. doi: 10.1371/journal.ppat.1011139 (PMC10249901; doi:10.1371/journal.ppat.1011139)

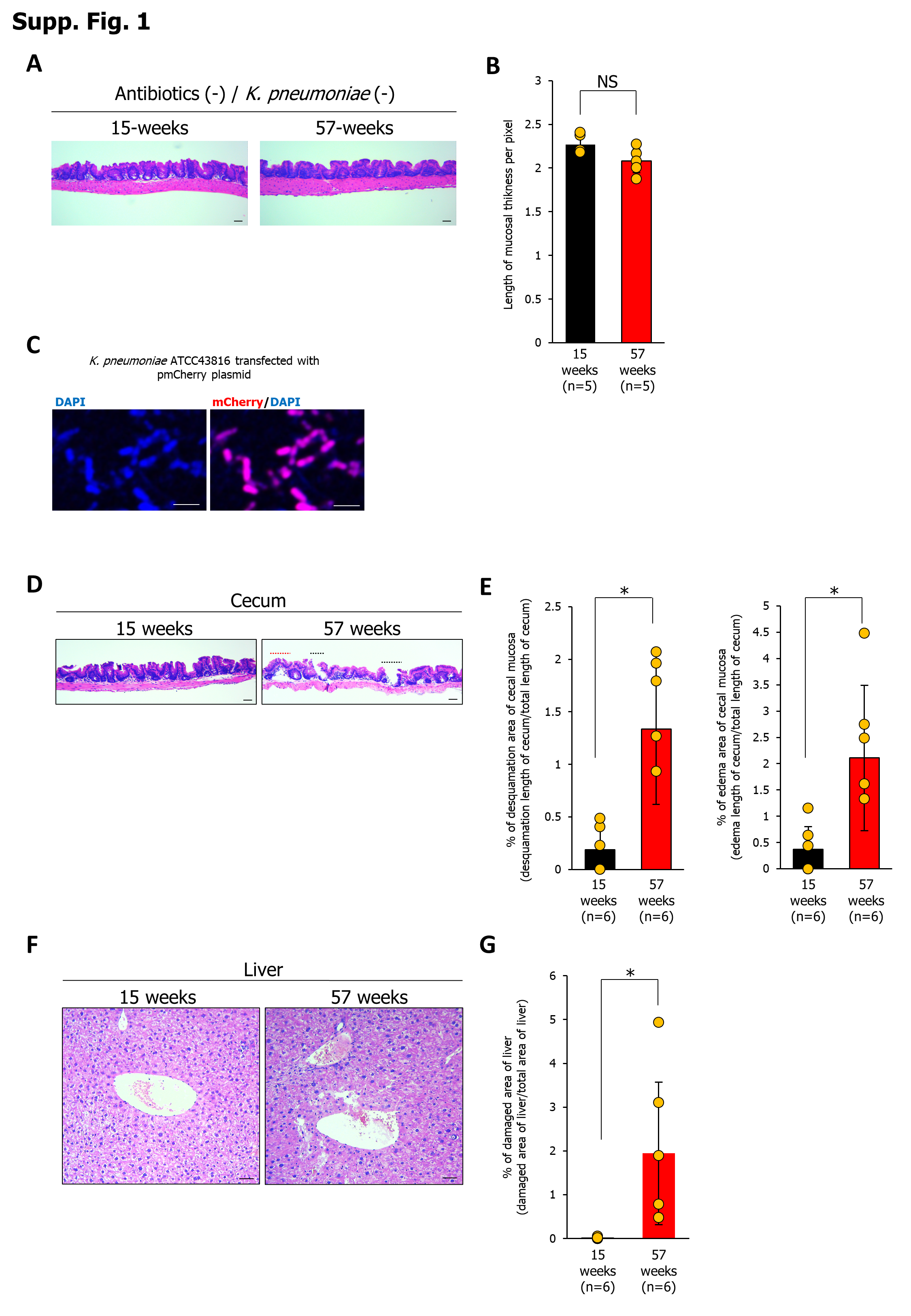

Supplement: S1 Fig — (A) H&E staining of cecal mucosa of 15- or 57-week-old mice before initiation of the antibiotic treatment. This image has five independent replicates. Scale bar = 50 μm. (B) Mucosal thickness was measured by ImageJ analysis software. Each dot represents the value from an individual mouse (n = 5 per group). Data are presented as the mean ± SD. NS: not significant. (C) K. pneumoniae ATCC43816 were electroporated with the pmCherry plasmid. This image has seven independent replicates. Scale bar = 5 μm. (D and F) H&E staining of cecum (D) and liver (F) tissue from K. pneumoniae ATCC43816 pmCherry-infected mice aged 15 or 57 weeks. Desquamation of cecal epithelium is denoted by the black dotted line, and edema of the cecal submucosa is indicated by the red dotted line. Each image has six independent replicates. Scale bar = 50 μm. (E) Desquamation and edema length of cecal mucosa was measured by the ImageJ analysis software, and the percentage of desquamation or edema area were calculated. Each dot represents the value from an individual mouse (n = 6 per group). Data are presented as the mean ± SD. *p < 0.05. p values were calculated by the Student’s t test. (G) Damaged area of liver per total area was measured and percentage calculated by ImageJ analysis software. Each dot represents the value from an individual mouse (n = 6 per group). Data are presented as the mean ± SD. *p < 0.05. p values were calculated by the Student’s t test. (TIF) [file ppat.1011139.s001.tif]

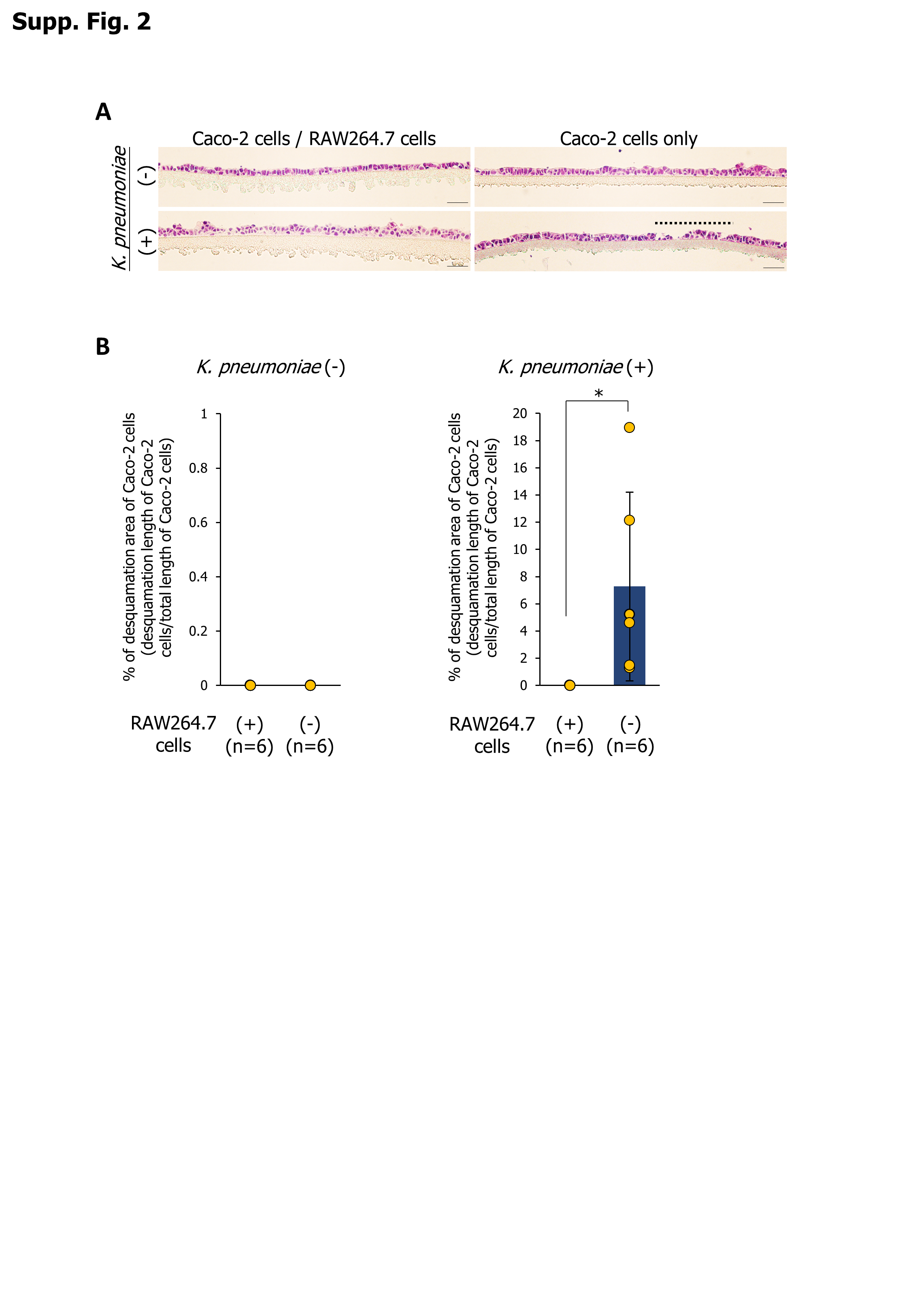

Supplement: S2 Fig — (A) H&E staining of K. pneumoniae-infected Caco-2 cells grown on the insert in the presence or absence of RAW264.7 macrophages. The cytoplasmic rupture of Caco-2 cells is indicated by the black dotted line. Each image has six independent replicates. Scale bar = 50 μm. (B) Desquamation length of Caco-2 cells grown on the insert was measured by the ImageJ analysis software and the percentage of desquamation area were calculated. Each dot represents six independent replicates (n = 6 per group). Data are presented as the mean ± SD. *p < 0.05. p values were calculated by the Student’s t test. (TIF) [file ppat.1011139.s002.tif]

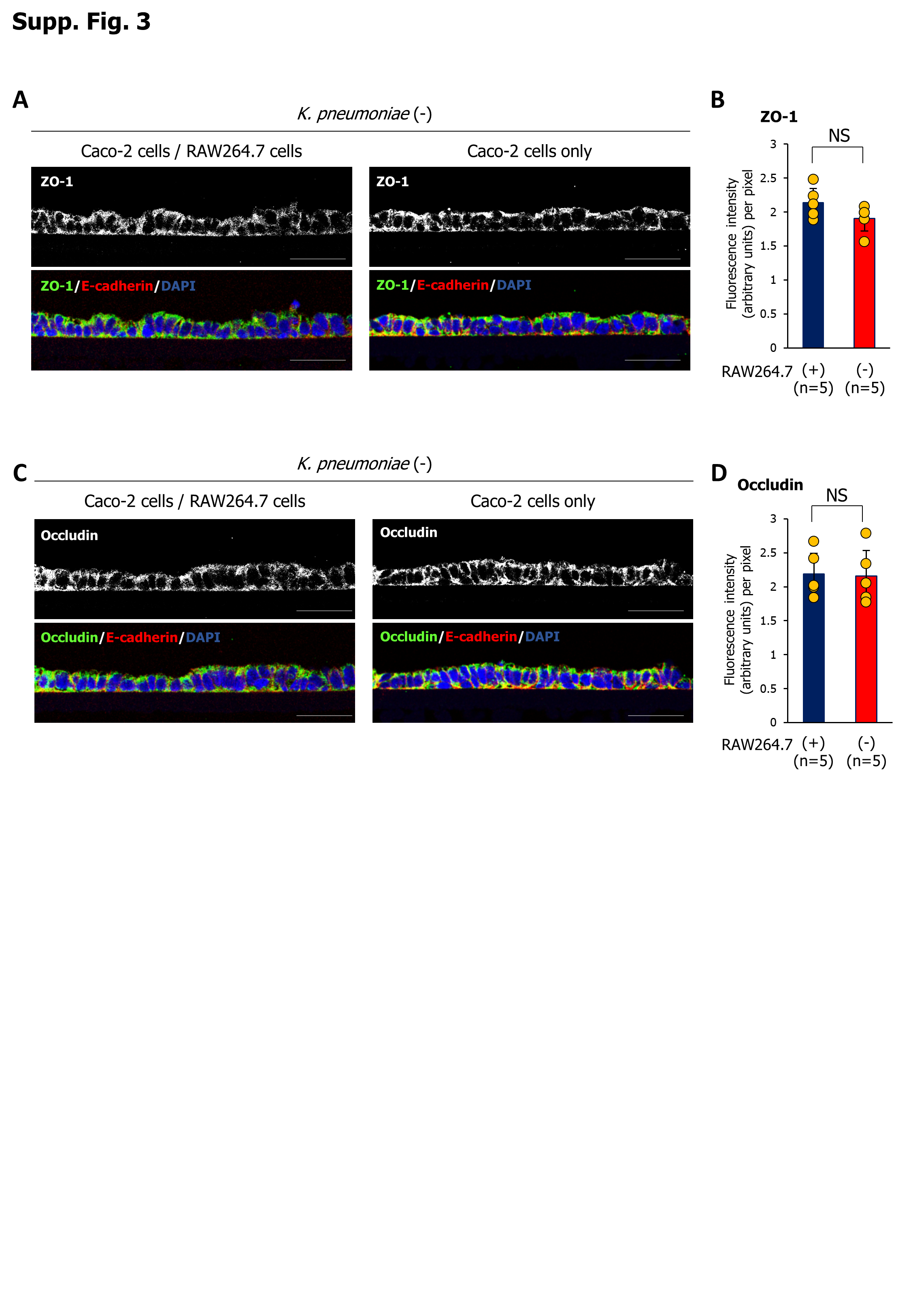

Supplement: S3 Fig — (A and C) Caco-2 cells grown on the insert in the presence or absence of RAW264.7 macrophages without K. pneumoniae infection were immunostained with an anti-E-cadherin antibody, and an anti-ZO-1 antibody (A) or an anti-occludin antibody (C). Each image has five independent replicates. Scale bar = 50 μm. (B and D) Fluorescence intensity per pixel of anti-ZO-1 antibody (B) or an anti-occludin antibody (D) were measured by the ImageJ analysis software. Each dot represents five independent replicates (n = 5 per group). Data are presented as the mean ± SD. NS: not significant. (TIF) [file ppat.1011139.s003.tif]

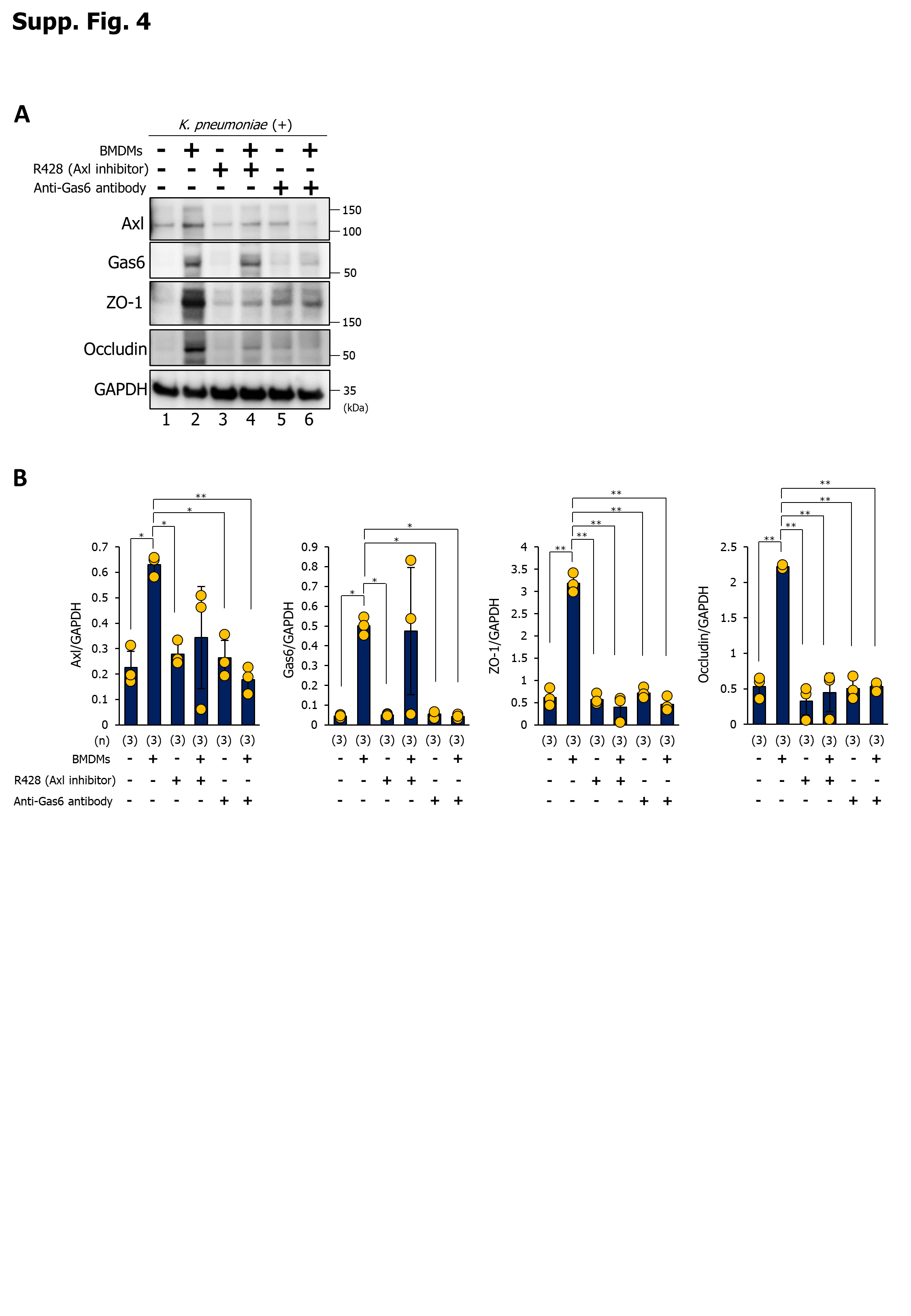

Supplement: S4 Fig — (A) K. pneumoniae infection model using a Transwell insert co-culture system based on Caco-2 cells and BMDMs were constructed. Prior to K. pneumoniae infection, Caco-2 cells were treated for 3 h with Axl inhibitor (R428; 20 nM) or 1 μg of anti-Gas6 antibody. Western blotting was performed to detect the expression of Axl, Gas6, ZO-1, and occludin in Caco-2 cells infected with K. pneumoniae in the presence of an Axl inhibitor (R428) or an anti-Gas6 antibody. Each western blotting image represents three independent replicates. (B) Western blotting signal intensity was analyzed by ImageJ software. Each dot represents three independent replicates (n = 3 per group). Data are presented as the mean ± SD. *p < 0.05, **p < 0.01. p values were calculated by one way analysis of variance. (TIF) [file ppat.1011139.s004.tif]

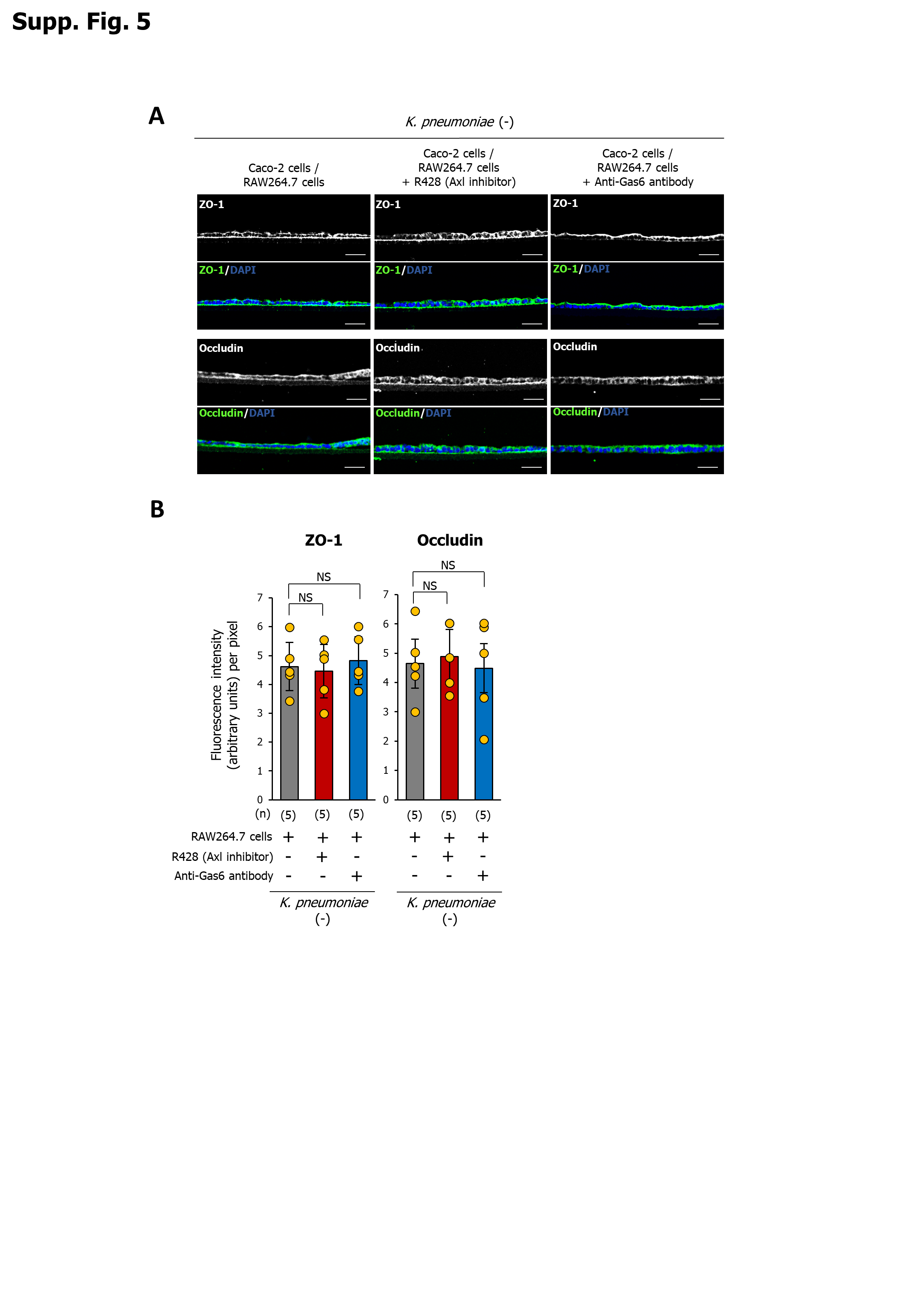

Supplement: S5 Fig — (A) Axl inhibitor (R428; 20 nM) or 1 μg of anti-Gas6 antibody were added to Caco-2 cells grown on the insert in the presence of RAW264.7 macrophages. Cells were immunostained with an anti-ZO-1 antibody and an anti-occludin antibody. Each image has five independent replicates. Scale bar = 50 μm. (B) Fluorescence intensity per pixel of anti-ZO-1 antibody or an anti-occludin antibody were measured by ImageJ analysis software. Each dot represents five independent replicates (n = 5 per group). Data are presented as the mean ± SD. NS: not significant. (TIF) [file ppat.1011139.s005.tif]

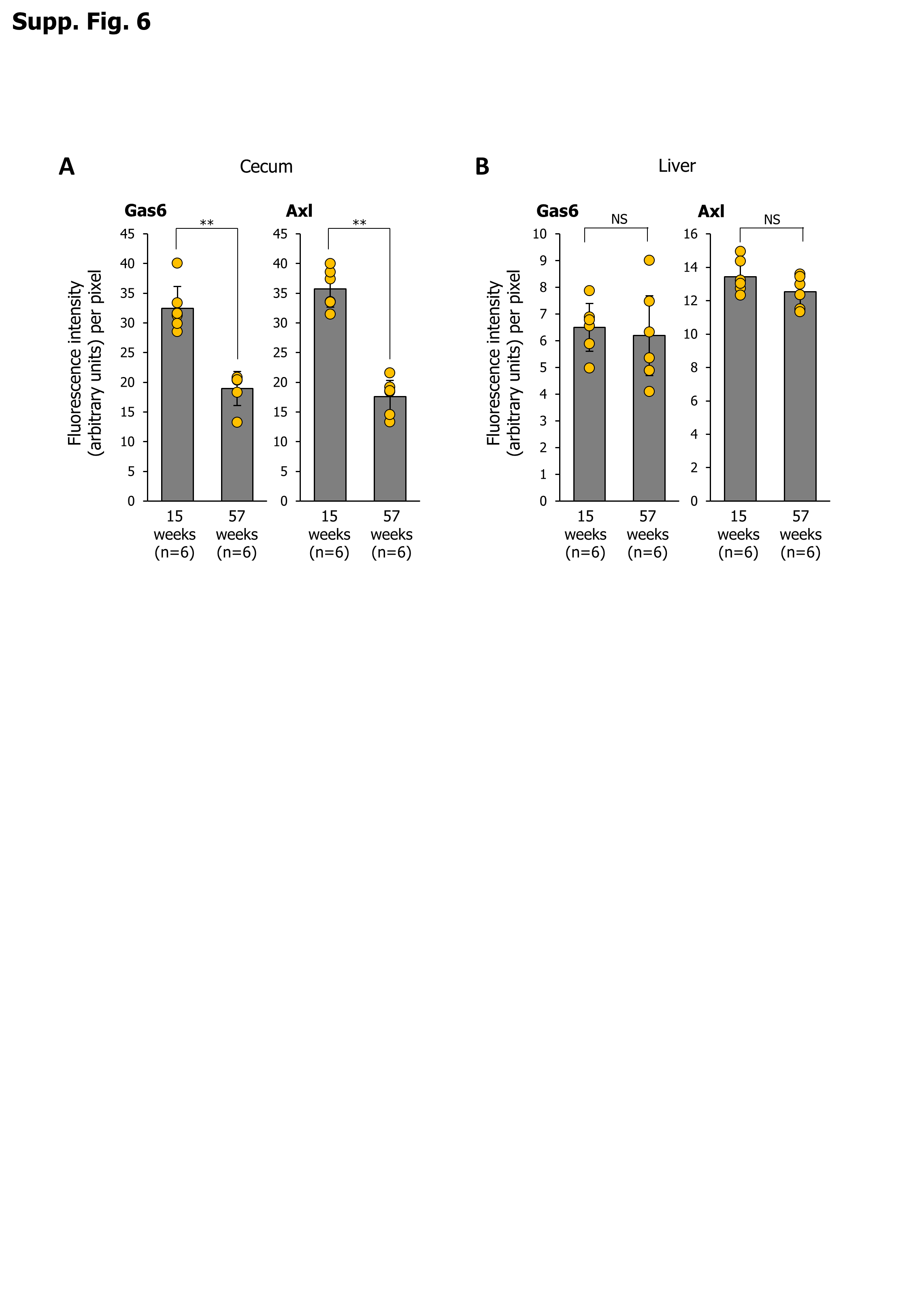

Supplement: S6 Fig — (A and B) Fluorescence intensity per pixel of anti-Gas6 or anti-Axl antibody of cecum (A) and liver (B) were measured by ImageJ analysis software. Each dot represents the value from an individual mouse (n = 6 per group). Data are presented as the mean ± SD. NS: not significant. ** p < 0.01. p values were calculated by the Student’s t test. (TIF) [file ppat.1011139.s006.tif]

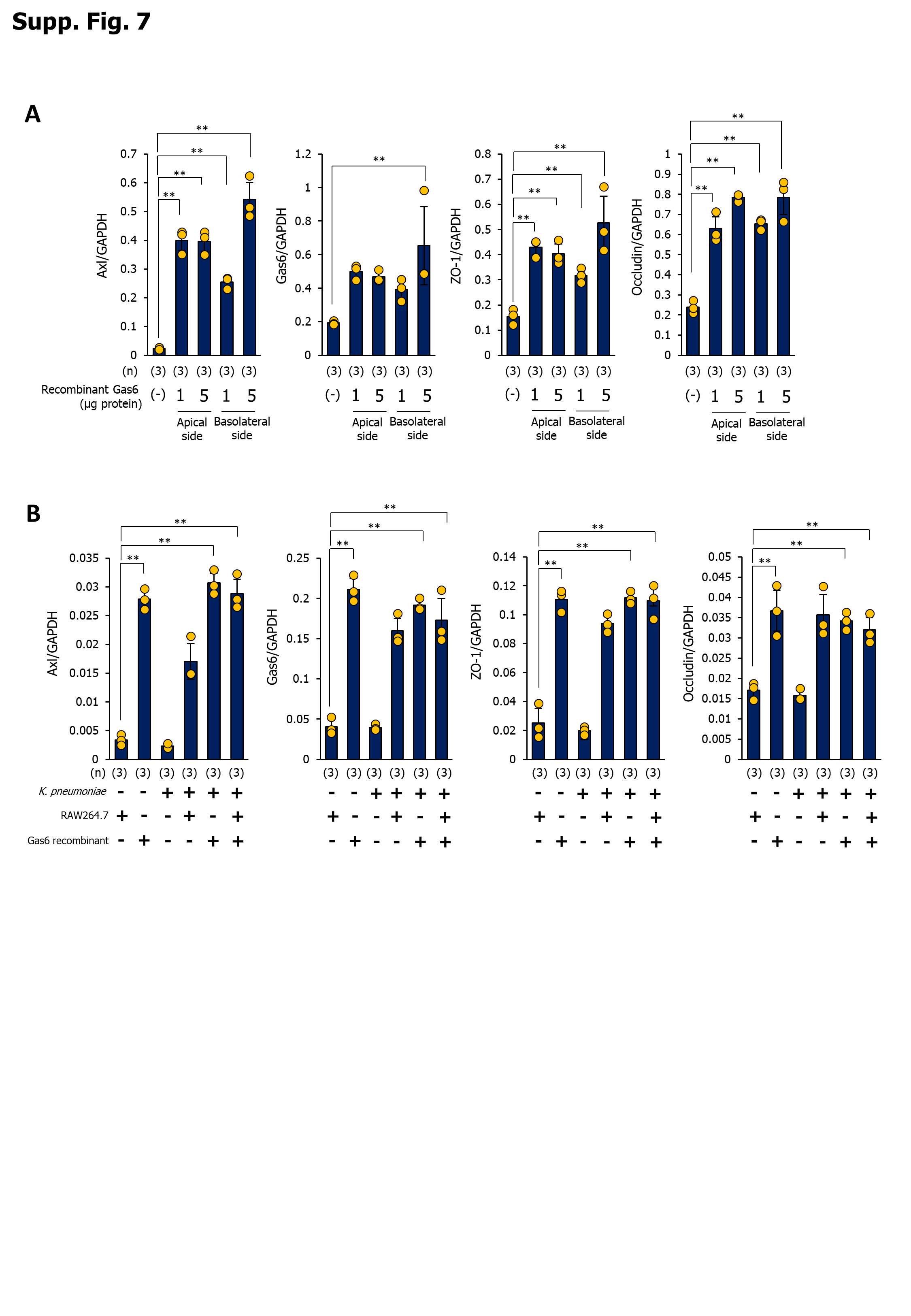

Supplement: S7 Fig — (A and B) Western blotting signal intensity (Fig 6B and 6C) was analyzed by ImageJ software. Each dot represents three independent replicates (n = 3 per group). Data are presented as the mean ± SD. **p < 0.01. p values were calculated by one way analysis of variance. (TIF) [file ppat.1011139.s007.tif]

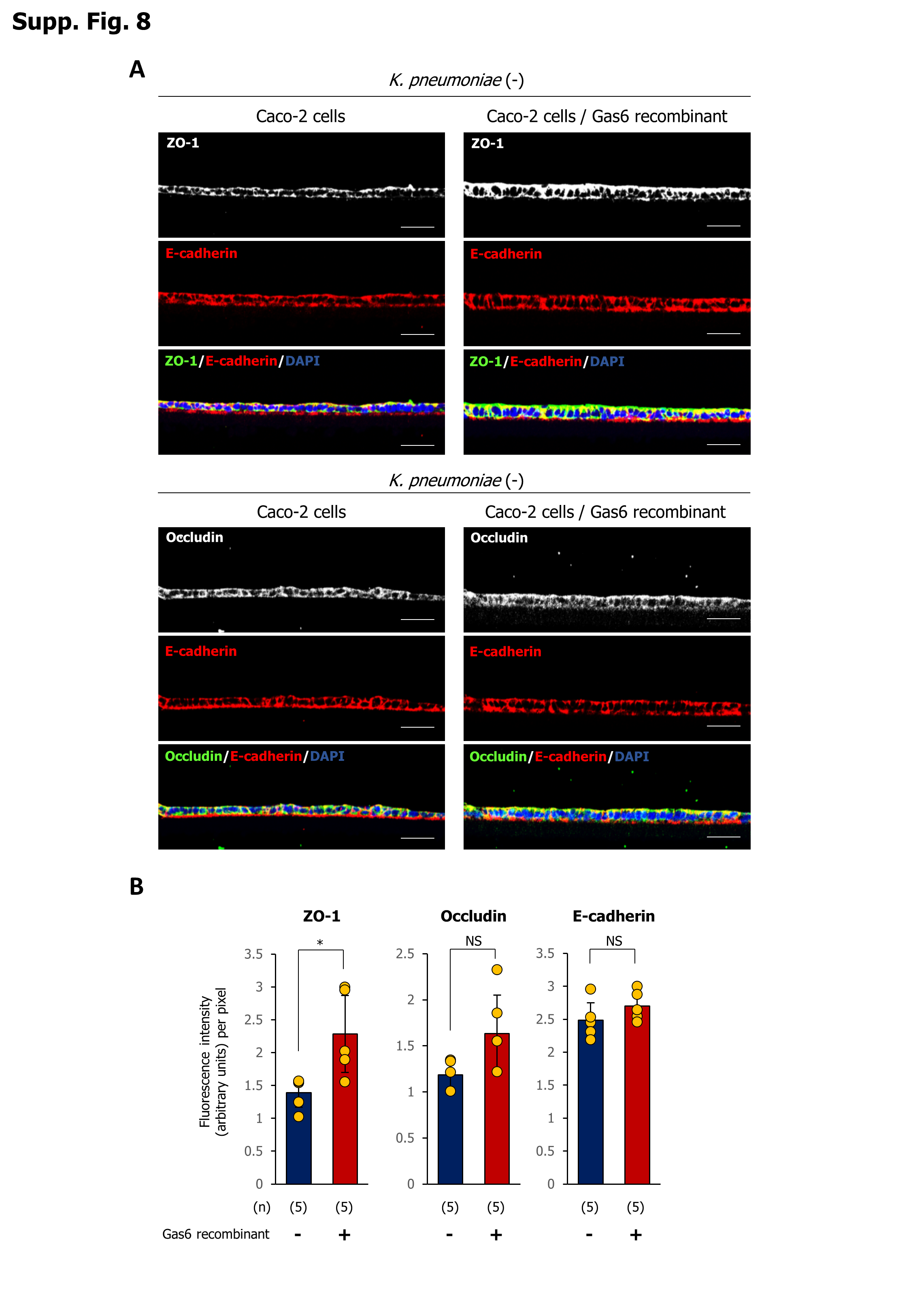

Supplement: S8 Fig — (A) Gas6 recombinant protein (1 μg) was added to Caco-2 cells grown on the Transwell insert for 3 h. Cells were immunostained with an anti-ZO-1 antibody and an anti-occludin antibody. Each image has five independent replicates. Scale bar = 50 μm. (B) Fluorescence intensity per pixel of anti-ZO-1 antibody, an anti-occludin antibody, and anti-E-cadherin antibody were measured by the ImageJ analysis software. Each dot represents five independent replicates (n = 5 per group). Data are presented as the mean ± SD. NS: not significant. *p < 0.05. p values were calculated by the Student’s t test. (TIF) [file ppat.1011139.s008.tif]

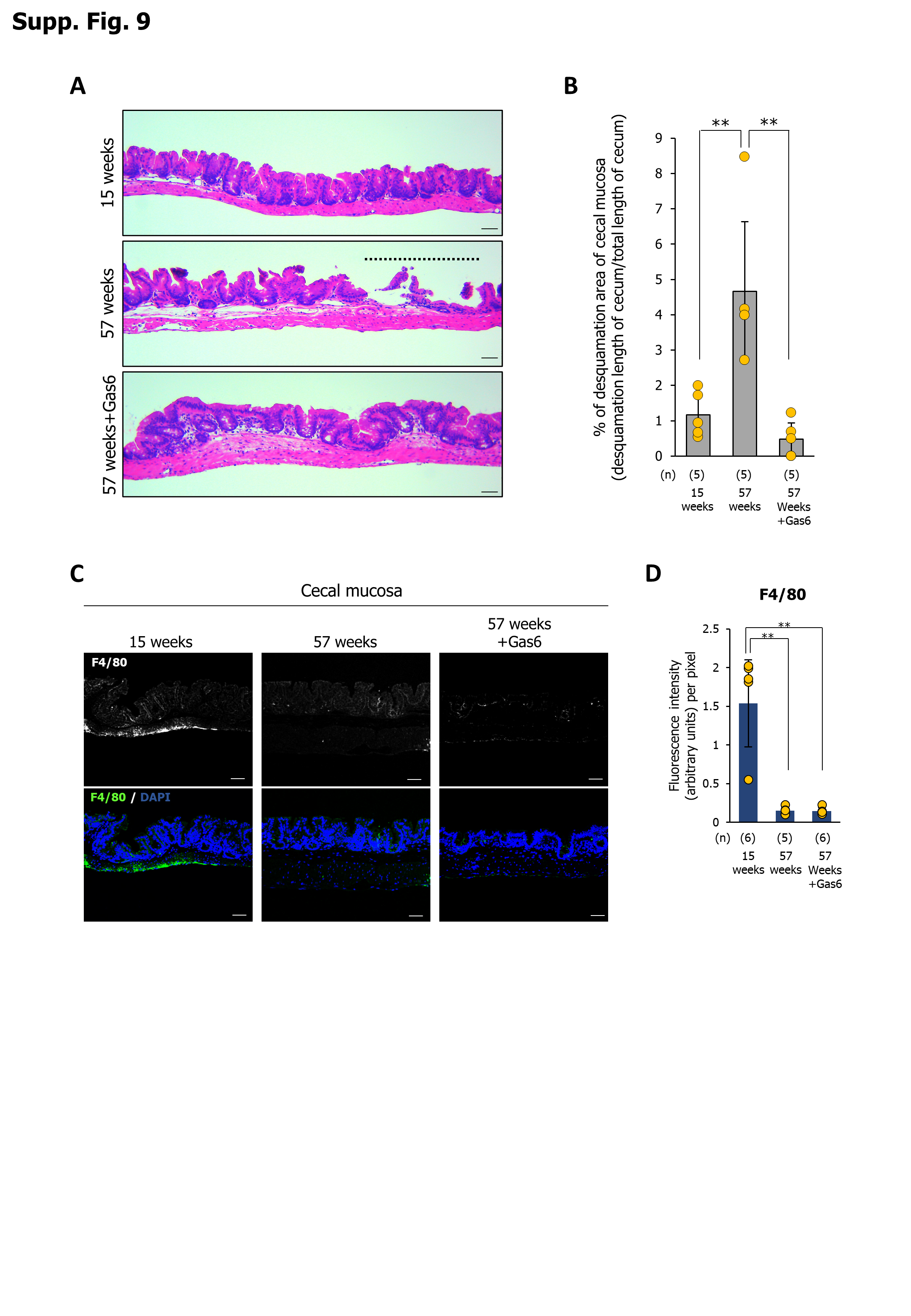

Supplement: S9 Fig — (A) H&E staining of cecum tissues from K. pneumoniae ATCC43816 pmCherry-infected mice. Desquamation of epithelial cells is indicated by the black dotted line. Each image has five independent replicates. Scale bar = 50 μm. (B) The area of desquamation was measured by ImageJ software. Each dot represents the value from an individual mouse (n = 5 per group). Data are presented as the mean ± SD. **p < 0.01. p values were calculated by one way analysis of variance. (C and D) Sections of cecal mucosa were from mice aged 15-week, 57-week, or Gas6 administered 57-week-old mice at 2 days after infection with K. pneumoniae ATCC43816 pmCherry and immunostained with an anti-F4/80 antibody. Each image has five or six independent replicates. Scale bar = 50 μm. Fluorescence intensity was analyzed by ImageJ software (D). Each dot represents the value from an individual mouse (n = 5 or 6 per group). Data are presented as the mean ± SD. **p < 0.01. p values were calculated by one way analysis of variance. (TIF) [file ppat.1011139.s009.tif]
