# Supplementary material for: Gas6 ameliorates intestinal mucosal immunosenescence to prevent the translocation of a gut pathobiont, Klebsiella pneumoniae, to the liver
Source: PLoS Pathog. 2023 Jun 8;19(6):e1011139. doi: 10.1371/journal.ppat.1011139 (PMC10249901; doi:10.1371/journal.ppat.1011139)
Supplement: S10 Fig — (PDF) [file ppat.1011139.s010.pdf]

Fig. 4A

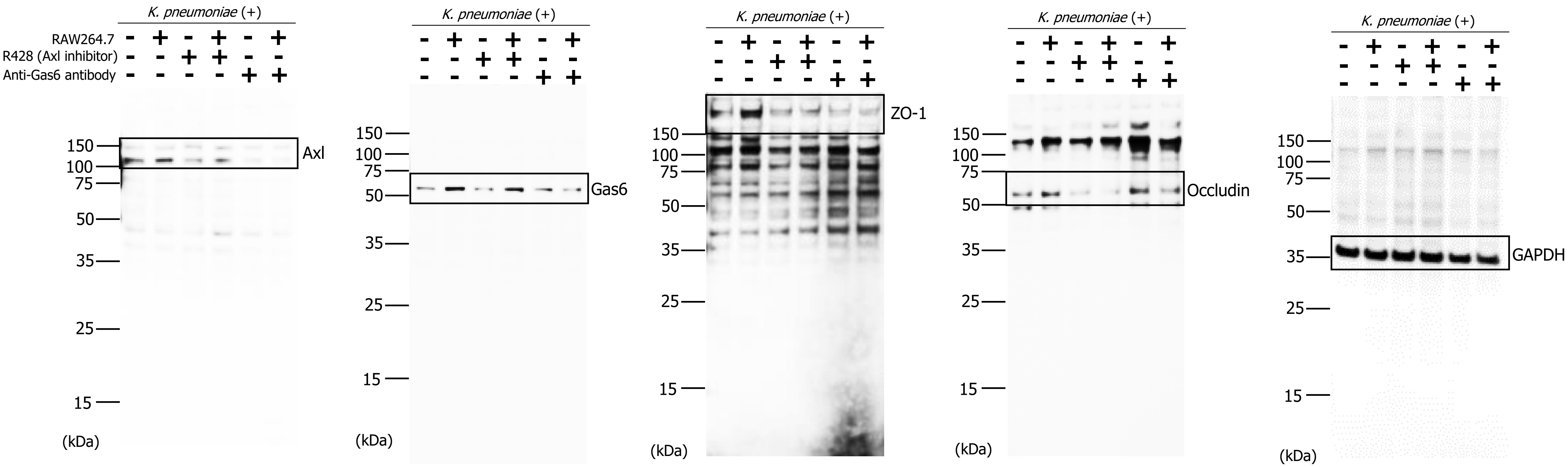
K. pneumoniae (+)
RAW264.7
R428 (Axl inhibitor)
Anti-Gas6 antibody
- + - + - +
- - + + - -
- - - - + +
150
100
75
50
35
25
15
(kDa)
Axl
Gas6
ZO-1
Occludin
GAPDH

Fig. 5C

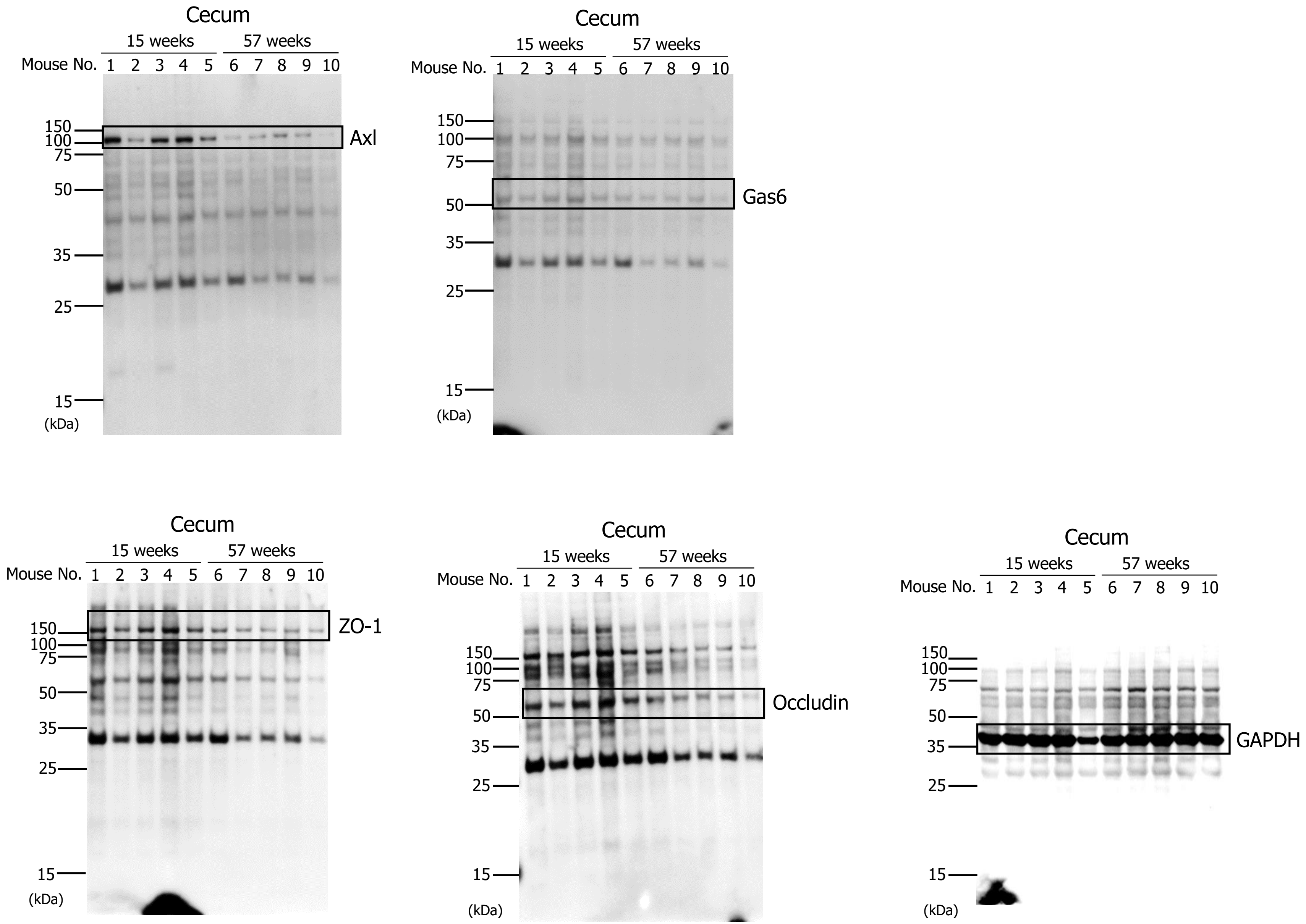
Cecum
15 weeks
57 weeks
Mouse No.
1 2 3 4 5 6 7 8 9 10
150
100
75
50
35
25
15
(kDa)
Axl
Gas6
ZO-1
Occludin
GAPDH

Fig. 5C

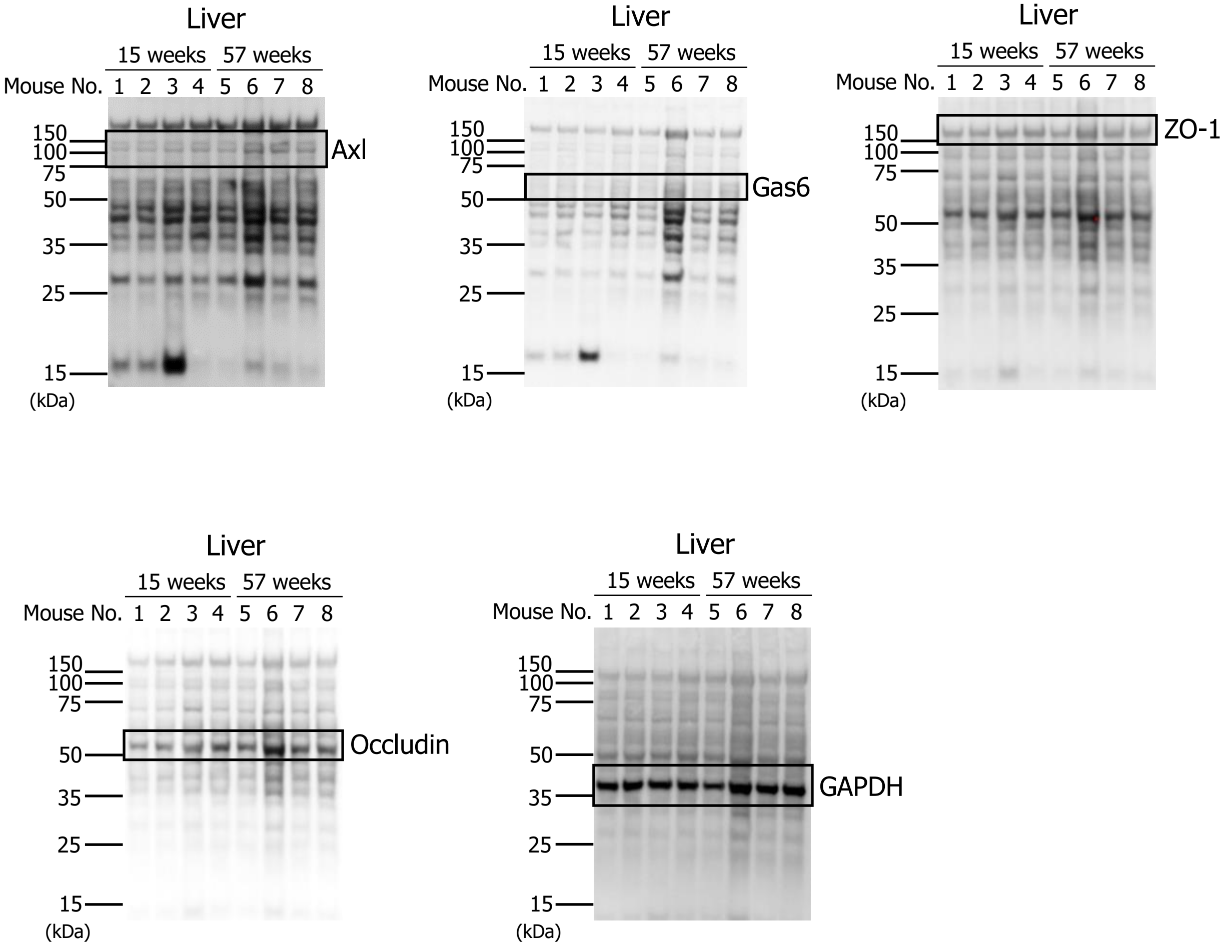
Liver
15 weeks
57 weeks
Mouse No. 1 2 3 4 5 6 7 8
150
100
75
50
35
25
15
(kDa)
Axl
Gas6
ZO-1
Occludin
GAPDH

Fig. 6B

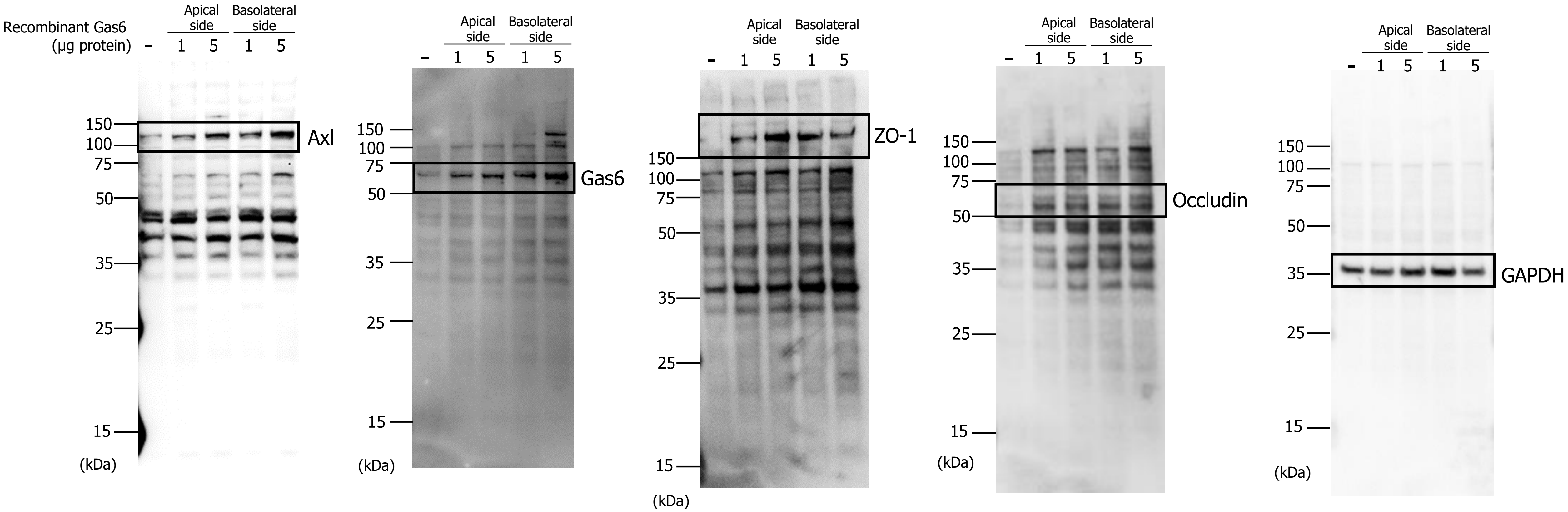

Recombinant Gas6
(μg protein)
Apical side
Basolateral side
- 1 5 1 5
150
100
75
50
35
25
15
(kDa)
Axl
Gas6
ZO-1
Occludin
GAPDH

Fig. 6C

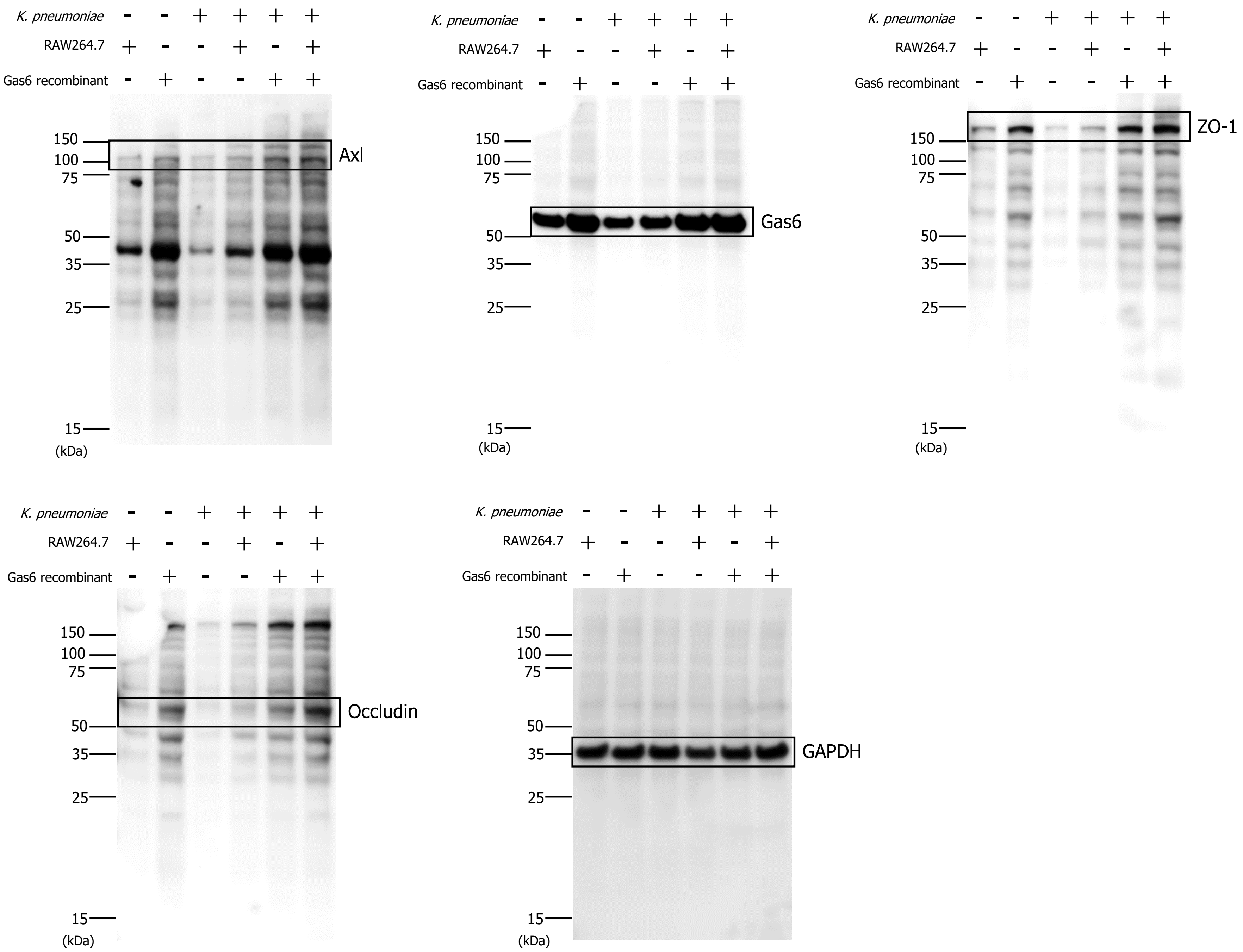
K. pneumoniae - - + + + +
RAW264.7 + - - + - +
Gas6 recombinant - + - - + +
150
100
75
50
35
25
15
(kDa)
Axl
Gas6
ZO-1
Occludin
GAPDH

Fig. 7D

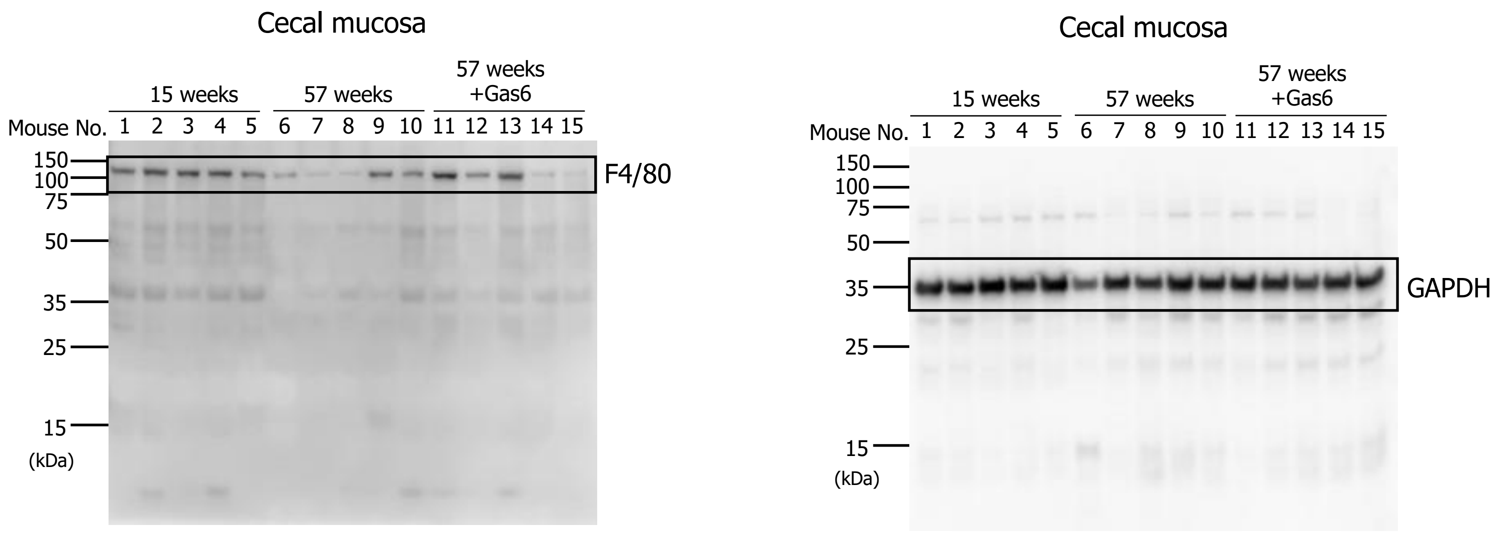
Cecal mucosa
15 weeks
57 weeks
57 weeks +Gas6
Mouse No. 1 2 3 4 5 6 7 8 9 10 11 12 13 14 15
150
100
75
50
35
25
15
(kDa)
F4/80
Cecal mucosa
15 weeks
57 weeks
57 weeks +Gas6
Mouse No. 1 2 3 4 5 6 7 8 9 10 11 12 13 14 15
150
100
75
50
35
25
15
(kDa)
GAPDH

Fig. 7H

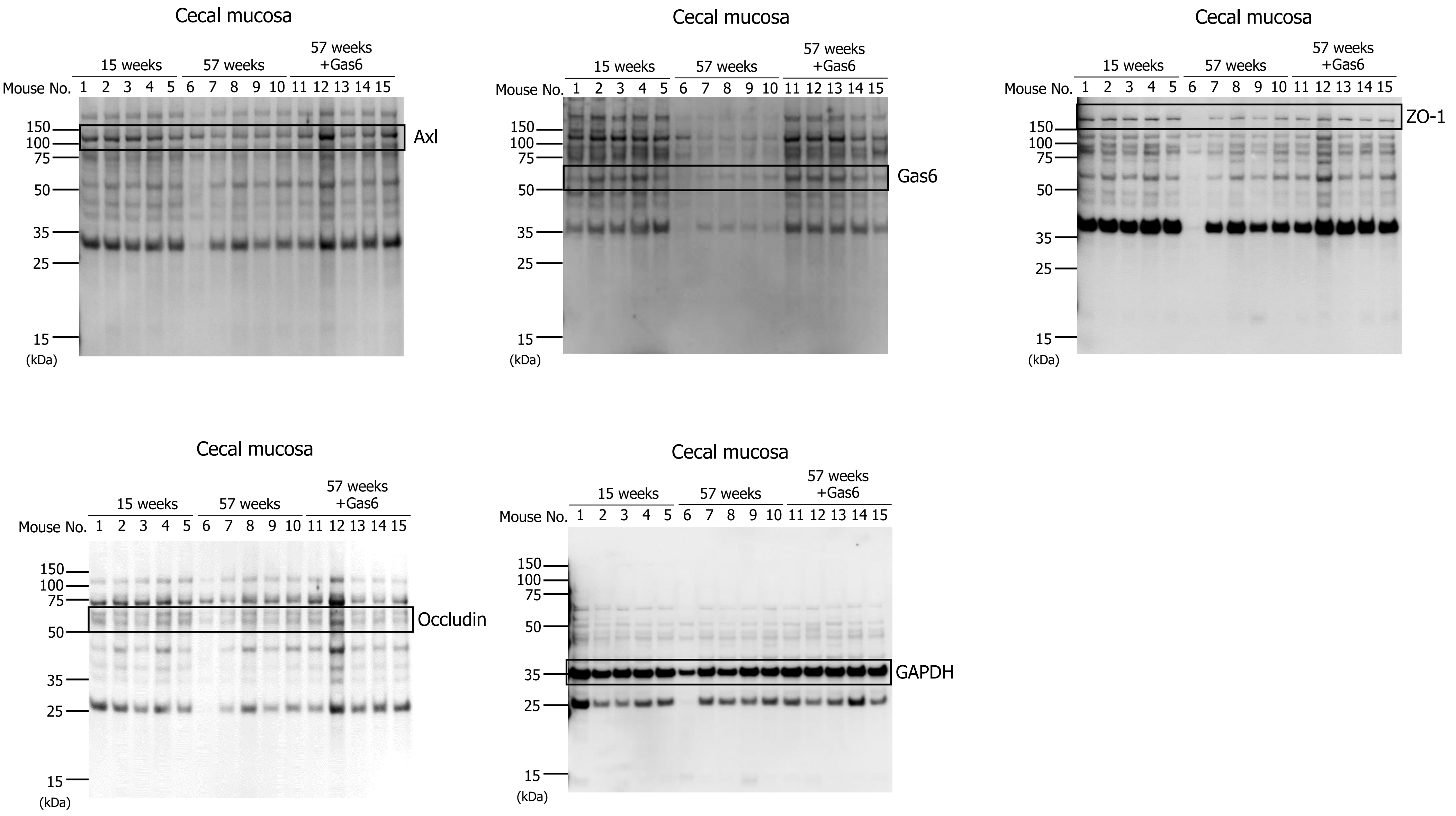
Cecal mucosa
15 weeks
57 weeks
57 weeks +Gas6
Mouse No. 1 2 3 4 5 6 7 8 9 10 11 12 13 14 15
150
100
75
50
35
25
15
(kDa)
Axl
Cecal mucosa
15 weeks
57 weeks
57 weeks +Gas6
Mouse No. 1 2 3 4 5 6 7 8 9 10 11 12 13 14 15
150
100
75
50
35
25
15
(kDa)
Gas6
Cecal mucosa
15 weeks
57 weeks
57 weeks +Gas6
Mouse No. 1 2 3 4 5 6 7 8 9 10 11 12 13 14 15
150
100
75
50
35
25
15
(kDa)
ZO-1
Cecal mucosa
15 weeks
57 weeks
57 weeks +Gas6
Mouse No. 1 2 3 4 5 6 7 8 9 10 11 12 13 14 15
150
100
75
50
35
25
15
(kDa)
Occludin
Cecal mucosa
15 weeks
57 weeks
57 weeks +Gas6
Mouse No. 1 2 3 4 5 6 7 8 9 10 11 12 13 14 15
150
100
75
50
35
25
15
(kDa)
GAPDH

Supp. Fig. 4

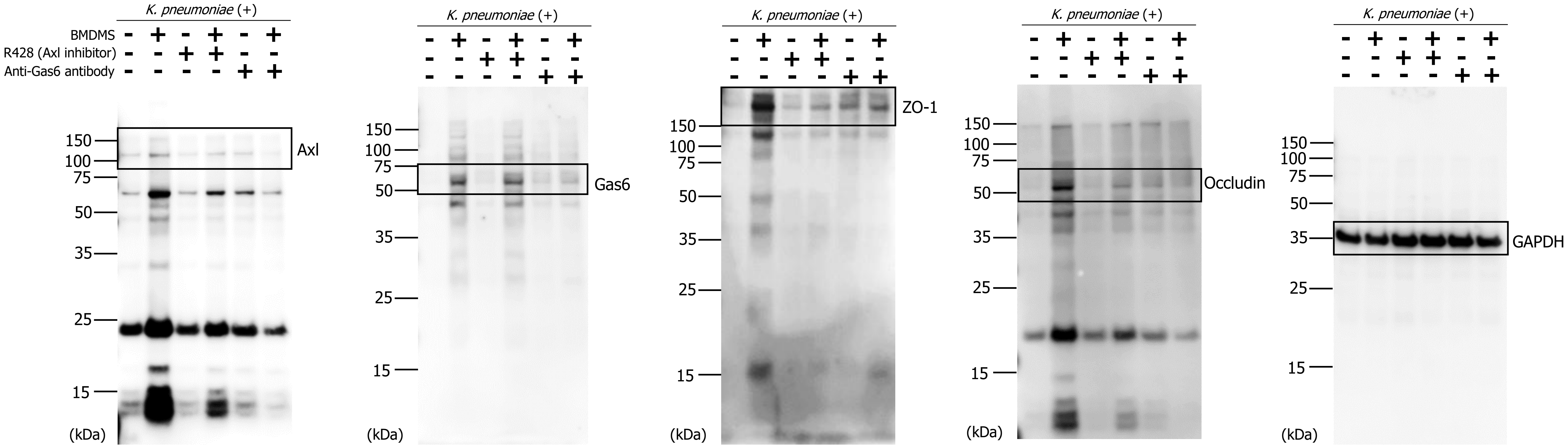
K. pneumoniae (+)
BMDMS
R428 (Axl inhibitor)
Anti-Gas6 antibody
Axl
Gas6
ZO-1
Occludin
GAPDH
150
100
75
50
35
25
15
(kDa)
