# Supplementary material for: Gas6 ameliorates intestinal mucosal immunosenescence to prevent the translocation of a gut pathobiont, Klebsiella pneumoniae, to the liver
Source: PLoS Pathog. 2023 Jun 8;19(6):e1011139. doi: 10.1371/journal.ppat.1011139 (PMC10249901; doi:10.1371/journal.ppat.1011139)
Supplement: Attachment — Submitted filename: a_point_by_point_response_PLoS_Pathogens_upload.docx [file ppat.1011139.s012.docx]

**A point-by-point response to each of the reviewers’ comments**

**Part I - Summary**

**Reviewer #1:** *In this manuscript, Tsugawa and colleagues describe a multidisciplinary study of the role of Gas6 in the translocation of Klebsiella pneumoniae (Kp) from the gut to the periphery, specifically the liver. The authors use an aged mouse system to model the immunosenescence hypothesized to contribute to the susceptibility of elderly patients to Kp infection. The data are presented clearly and logically, and the authors deserve credit for the creative use of their aged mouse system. That said, there are several areas where the study and manuscript can be improved.*

**(Response)**

We appreciate the reviewer’s critical comments. We have taken specific comments from the reviewer into account in the revised manuscript, and we conducted several additional experiments. In the revised version of the manuscript, we have heeded all advice suggested and amended the manuscript accordingly. Detailed responses are provided below.

**Reviewer #2:**

*In the manuscript, Gas6 ameliorates intestinal mucosal immunosenescence to prevent the translocation of a gut pathobiont, Klebsiella pneumoniae, to the liver" Tsugawa and colleagues combine infection experiments in antibiotic treated young and aged mice and an cell-culture based assay to study the impact of the protein Gas6 on the gut pathogen K. pneumoniae. They demonstrate that Gas6, which is released by intestinal macrophages, can recognize K. pneumoniae and thereby inhibits bacterial translocation through tight-junction barrier enhancement. As the amount of secreting macrophages decreases with age and administration of recombinant protein reverted the phenotype, the authors conclude that reduced Gas6 levels increase the susceptibility against K. pneumoniae in elderly mice. I think the findings are interesting and relevant to experts in the field. I think the manuscript is well written and generally convincing, although I do have some questions that I outline below, specifically regarding the performed animal experiments.*

**(Response)**

We appreciate these critical comments and insightful suggestions, which have helped us to considerably improve our paper. In the revised version of the manuscript, we have heeded all advice suggested and amended the manuscript accordingly. Detailed responses are provided below.

**Part II – Major Issues: Key Experiments Required for Acceptance**

**Reviewer #1:**

**Comment 1.** *The use of strain ATCC43816 makes sense in that it is the most well-characterized Kp lab strain; however, this is a hypervirulent strain. Hypervirulent strains garner attention due to their ability to infect younger, healthy individuals. Although elderly individuals are also infected by hypervirulent strains, it would benefit this study to validate the authors' findings with a non-hypervirulent strain. These strains are more prevalent in healthcare settings, especially those with an elderly population.*

**(Response)**

Thank you very much for this important comment. Since the virulence of *K. pneumoniae* is known to differ among the bacterial strains and there are variety of virulence factors to determine the pathogenicity, it certainly is important study to determine whether Gas6 prevents the bacterial invasion and translocation from gastrointestinal tract to the liver even against non-hypervirulent strain infections. In this study, we show that Gas6 secreted by macrophage inhibits the invasion of *K. pneumoniae* ATCC43816 into the intestinal epithelium by increasing expression of tight-junction proteins. This increased expression is a Gas6-dependent host response to *K. pneumoniae* infection, and therefore Gas6 would be expected to prevent bacterial invasion regardless of the strain. On the other hand, further studies are needed to determine whether the inducibility of Gas6 secretion from macrophages differs among the strains. We will collect several *K. pneumoniae* strains, including non-hypervirulent strains, and will examine the preventive effects of Gas6 on each *K. pneumoniae* strain infection. These important points have been specifically addressed in Discussion sections. The revised texts that are changed the color within the manuscript are extracted, as follow:

**Line 290**: *K. pneumoniae* ATCC43816 is a well-studied hypervirulent laboratory strain which has K2 serotype capsules and can cause an acute respiratory disease in animal models (***PLoS One***, 9: e107394, 2014). Recently, genomic sequencing analysis revealed that *K. pneumoniae* ATCC43816 possess the known virulence determinant for a siderophore-dependent iron uptake system (yersiniabactin), a Type 4 secretion system, and a CRISPR system, which are absent from non-hypervirulent strains (***PLoS One***, 9: e107394, 2014). In this study, we show that Gas6 inhibits the invasion of *K. pneumoniae* ATCC43816 to the intestinal epithelium by enhancing tight-junction barriers, contributing to the prevention of the bacterial translocation. Since hypervirulent strains have the ability to infect young, healthy individuals, the activity of Gas6 in preventing the bacterial translocation of hypervirulent strains is beneficial information to prevent *K. pneumoniae* infection.

*K. pneumoniae* is reported to invade human epithelial cell lines. The invasion rates among each clinically isolated strain range from 10.1 – 48.6%, indicating that invasive abilities are differ among the bacterial strains (***Infection and immunity***, 65:4445–4451, 1997, ***Infection and immunity***, 65:2950-2958, 1997). The difference in invasiveness is reported to not be dependent on capsule type, suggesting that invasion abilities are mediated by multifactorial process and that capsule alone is insufficient to account for differences in pathogenicity between strains (***Infection and immunity***, 65:4445–4451, 1997). Pan *et al*. also reported that additional genetic loci, including genes for LPS, the ClpX protease, and carnitine metabolism, contribute to the virulence of *K. pneumoniae* (***Infection and immunity***, 79:997-1006, 2011). From these observations, it was thought that virulence factors of *K. pneumoniae* are diverse and it may be difficult to identify the factors which determined the difference in virulence among the bacterial strains. The protective ability of Gas6 to the invasion of *K. pneumoniae* depends on the Gas6/Axl signaling in epithelial cells, and therefore Gas6 can be inferred to prevent the bacterial invasion regardless of the bacterial strain. Further studies are needed to determine whether Gas6 also prevents the bacterial translocation from the gastrointestinal tract against non-hypervirulent *K. pneumoniae* strains, which are particularly prevalent in the elderly, by analyzing the inducibility of Gas6 secretion from macrophages by several strains.

**Comment 2.** *Similarly, the in vitro data is generated entirely with the RAW264.7 and Caco-2 cell lines. Given that these cell lines are immortalized, this study would benefit from validation with primary cells, either mouse or human.*

**(Response)**

Thank you for the comments. In accordance with your comments, we constructed *in vitro* infection models using murine bone marrow-derived macrophages (BMDMs) as primary macrophage cells isolated from young mice instead of RAW264.7 cells and conducted several additional experiments. First, as shown in revised **Fig. 2C**, the number of bacteria invading Caco-2 cells was significantly increased in the absence of BMDMs compared with that in the presence of BMDMs. We next examined whether the inhibition of *K. pneumoniae* invading Caco-2 cells by BMDMs is due to the induction of ZO-1 and/or occludin expression in Caco-2 cells by Gas6 secreted from BMDMs. As shown in revised **Fig S4**, Caco-2 cells were treated with an Axl inhibitor (R428; 20 nM) or 1 μg of anti-Gas6 antibody for 3 h prior to infection with *K. pneumoniae*. Upon *K. pneumoniae* infection, the expression of Axl, Gas6, ZO-1, and occludin in Caco-2 cells was increased by the presence of BMDMs (**S4A Fig, lanes 1 and 2, and S4B Fig**). The increased expression levels of Axl, ZO-1, and occludin in Caco-2 cells was significantly reduced by Axl inhibitor (R428) or anti-Gas6 antibody (**S4A Fig, lanes 4 and 6, and S4B Fig**). Additionally, the inhibitory effects of the bacterial invasion within Caco-2 cells by BMDMs was abolished upon treatment with an Axl inhibitor or anti-Gas6 antibody (**Fig 4G**). These results revealed that the inhibitory effects of the bacterial invasion within Caco-2 cells by Gas6 can also be evaluated with *in vitro* infection models using primary macrophages. In the future, we need to examine whether the Gas6 secretory response against *K. pneumoniae* is changed by aging using BMDMs isolated from elderly mice.

Recently, host–microbe interaction studies using intestinal organoid cultures have advanced and shown great promise (***Current Opinion in Immunology***, 48, 15-22, 2017). Additionally, triple co-culture systems of organoid, immune cells, and bacteria are reported to be a futuristic next step (***Current Opinion in Immunology***, 48, 15-22, 2017). Then, we have now begun to construct an *in vitro* infection model using co-cultures systems of intestinal organoids and BMDMs. However, in this model, intestinal organoids are required to culture a two-dimensional monolayer, and therefore substantial preliminary study is needed. Once we have established this *in vitro* infection model, we plan to conduct further studies to determine the detailed mechanisms controlling the inhibitory effects of Gas6 against *K. pneumoniae* infection by using co-cultures systems of intestinal organoids and BMDMs. Again, thank you very much for the important comments. These data are shown in Figure 2C, Figure S4 and Figure 4G of the revised manuscript, and the Results, Discussion, and Methods sections have been revised to include these data. The revised texts that are changed the color within the manuscript are extracted, as follow:

**Line 140**: Furthermore, in the absence of RAW264.7 or BMDMs, the number of bacteria invading Caco-2 cells was significantly higher than that in the presence of RAW264.7 or BMDMs, suggesting that macrophages inhibit bacterial invasion into epithelial cells (**Fig 2B and 2C**).

**Line 182**: Upon *K. pneumoniae* infection, the expression of Axl, Gas6, ZO-1, and occludin in Caco-2 cells was significantly increased by the presence of RAW264.7 macrophages (**Fig 4A; lanes 1 and 2, and 4B**) or BMDMs (**S4A Fig; lanes 1 and 2, and S4B Fig**). Axl inhibitor (R428) or anti-Gas6 antibody significantly attenuated the increase in the expression of Axl, ZO-1, and occludin of Caco-2 cells in the presence of RAW264.7 macrophages (**Fig 4A; lanes 4 and 6, and 4B**) or the presence of BMDMs (**S4A Fig; lanes 4 and 6, and S4B Fig**).

**Line 196**: Additionally, the decreased invading bacterial number in the presence of BMDMs was eliminated by treatment with an Axl inhibitor or anti-Gas6 antibody (**Fig 4G**).

**Line 367**: Organoid cultures are known to be excellent platforms to examine host–microbe interaction mechanisms and triple co-culture systems of organoid, immune cells, and bacteria are reported to be a futuristic next step (***Current Opinion in Immunology***, 48, 15-22, 2017). By utilizing a triple co-culture system of intestinal organoids, BMDMs, and *K. pneumoniae*, it is expected that the detailed mechanisms of the interaction between *K. pneumoniae* and host epithelial cells will be revealed.

**Line 447**: BMDMs were prepared by flushing bone marrow from femurs and tibiae of 20-week-old mice with DMEM, and were cultured in DMEM supplemented with 10% FBS and 20 ng/mL recombinant mouse macrophage-colony stimulating factor (PeproTech, Rocky Hill, NJ, USA, cat. no. 315-02) for 7 days (***PLoS Biology***, 18(9): e3000813, 2020).

**Comment 3.** *The data shown in Fig. 7C demonstrate that treatment with recombinant Gas6 reduced bacterial load in the cecum. The authors do not explore this finding in any detail. Does Gas6 have direct bactericidal effects in vivo? Or does Gas6 stimulate macrophage migration to the intestinal mucosa and reverse the effects seen in Fig 5E? This is important to understand, as it is unclear if the data shown in 7B are due to the effects of Gas6 on gut tight junction formation, as is posited throughout the manuscript, or simply due to the reduction in bacterial load.*

**(Response)**

Thank you very much for this important comment. In accordance with your suggestion, we conducted several additional experiments. First, to examine whether Gas6 recombinant protein has direct bactericidal activity, the bacterial number in the cecal contents (mouse stool) were measured. The infected bacterial number in the cecal contents were not affected by the administration of Gas6 recombinant protein, suggesting that Gas6 does not directly reduce the infected bacterial load (**Fig 7C**). Second, to examine whether administration of Gas6 stimulate macrophage migration to the intestinal mucosa of 57-week-old mice, the expression levels of F4/80 were measured by western blotting and immunofluorescence analysis. The reduced expression levels of F4/80 of 57-week-old mice were not restored by Gas6 administration (**Fig 7D and 7E**). Additionally, the immunostaining analysis showed no increase in the staining signals of F4/80 in the cecal mucosa of 57-week-old mice upon the administration of Gas6 protein (**S9C and S9D Fig)**. From these results, it was not thought that Gas6 directly stimulate the migration of macrophages to the intestinal mucosa of elderly mice and restore the number of intestinal mucosal macrophages. Then, we analyzed the localization of Gas6 recombinant protein in the cecal mucosa of mice administered with Gas6. While colocalization of Gas6 and Axl in the cecal epithelium of 15-week-old mice was observed, the staining signals of Gas6 were scarcely detected in 57-week-old mice (**Fig 7F and 7G**). Administration of Gas6 recombinant protein in the 57-week-old mice restored the staining levels of Gas6 on the cecal epithelium and the colocalization of Gas6 and Axl was also detected (**Fig 7F and 7G**). Further, the expression levels of Axl, ZO-1, and occludin in the cecal mucosa of Gas6-treated 57-week-old mice infected with *K. pneumoniae* were restored to levels similar to those in 15-week-old mice (**Fig 7H and 7I**). From these findings, we concluded that Gas6 recombinant protein enhances the tight-junction barriers by stimulating Gas6/Axl signals in the intestinal epithelium, leading to exert the protective effect against *K. pneumoniae* infection in elderly hosts. These data are shown as Figure 7 and Supplementary Figure 9 of the revised manuscript, and the Results sections have been revised to include these data. The revised text that is changed the color within the manuscript is extracted, as follow:

**Line 262**: In addition, the administration of Gas6 did not reduce the infected bacterial number in the cecal contents, indicating that Gas6 does not directly reduce the infected bacterial load (**Fig 7C**). To next examine whether administration of Gas6 restores the number of intestinal mucosal macrophages in 57-week-old mice, the expression levels of F4/80 were measured by western blotting and immunofluorescence analysis. The expression levels and immunostaining signals of F4/80 in the cecal mucosa of *K. pneumoniae*-infected 57-week-old mice were significantly decreased compared with that of 15-week-old mice (**Fig 7D and 7E, and S9C and S9D Fig**). The reduced F4/80 expression levels of *K. pneumoniae*-infected 57-week-old mice were not restored by Gas6 administration (**Fig 7D and 7E, and S9C and S9D Fig**). We then analyzed the localization of Gas6 recombinant protein in the cecal mucosa of *K. pneumoniae*-infected mice administered with Gas6. While colocalization of Gas6 and Axl in the cecal epithelium of 15-week-old mice was observed, the staining signals of Gas6 were scarcely detected in 57-week-old mice (**Fig 7F and 7G**). Administration of Gas6 recombinant protein in the 57-week-old mice restored the staining levels of Gas6 on the cecal epithelium and the colocalization of Gas6 and Axl was also detected (**Fig 7F and 7G**). Further, the expression levels of Axl, ZO-1, and occludin in the cecal mucosa of Gas6-treated 57-week-old mice infected with *K. pneumoniae* were also restored to levels similar to those in 15-week-old mice (**Fig 7H and 7I**). These findings suggest that Gas6 recombinant protein enhances the tight-junction barriers in the intestinal epithelium by directly stimulating Gas6/Axl signals, leading to the protective effect being exerted against *K. pneumoniae* infection in elderly hosts.

**Comment 4.** *There are several areas where images lack quantification (such as what is shown in Fig. 2D, 5C, and/or S3B) and experimental information. These are S1B, S2, Fig 1D-E, 3D, 4A, 5A-B, and 6B-C. For all images, please indicate the number of replicates these images represent.*

**(Response)**

Thank you for this comment. We conducted quantification analysis for all images (western blotting and immunofluorescence) using the ImageJ analysis software and we added the experimental information including the number of replicates these images represent to the Methods sections and Figure Legends. Methods and Figure legends sections have been revised. The revised texts that are changed the color within the manuscript are extracted, as follow:

**Methods sections**

**Line 468**: In animal experiments, two independent experiments were performed on different days. The first experiment was performed to collect preliminary data and the second experiment was performed for data collection. The number of representative image replicates, and the number of each sample, is described within the relevant Figure legend. Blind analysis of the data was ensured by providing investigators with numbered images. Image data were saved as TIFF files. Images for which the analysis by ImageJ software showed average value results were selected as representative images.

**Line 524**: Fluorescence intensity of immunostaining was measured as intensity values per pixel using ImageJ analysis software (National Institute of Health, Bethesda, MD, USA).

**Line 530**: Western blotting signal intensity of each band was measured using ImageJ software. The number of representative image replicates and the number of each sample is described within Figure legend and Figure. Image data were saved as TIFF files, and images for which the image analysis by ImageJ analysis software results showed an average value were selected as representative images.

**Figure legends sections**

**Fig. 1: (A)** Treatment scheme used to analyze age-related differences in susceptibility to *K. pneumoniae*. Mice aged 15- or 57-week were administered antibiotics for 4 weeks prior to oral inoculation of *K*. *pneumoniae* ATCC43816 pmCherry (5 × 10^7^ bacteria). Survival was monitored daily (Experiment A). At 2 days post-infection, the mice were sacrificed, and the cecum and liver were harvested (Experiment B). **(B)** Effect of aging on the survival of mice infected with *K. pneumoniae* ATCC43816 pmCherry. Each dot was represented from an individual mouse (n = 6 per group). *p*-values were determined using the log-rank test. **(C)** Bacterial counts in the cecum and liver were determined 2 days after infection. Cecum and liver tissues were homogenized in PBS, and the homogenates were plated onto LB agar containing 400 μg/mL ampicillin. The number of CFU was determined. Each dot represents the value from an individual mouse (n = 5 per group). Data are presented as the mean ± SD. **p* < 0.05, ***p* < 0.01. *p* values were calculated by Student’s *t* test. **(D and F)** Sections of cecal mucosa (D) or liver (F) were from mice aged 15 or 57 weeks at 2 days after infection with *K. pneumoniae* ATCC43816 pmCherry and immunostained with an anti-mCherry antibody and rhodamine phalloidin staining. Each image has six independent replicates. Scale bar = 20 μm. **(E)** Number of immunostained puncta of *K. pneumoniae* within the cecal submucosa per same area were counted by the ImageJ analysis software. Each dot represents the value from an individual mouse (n = 6 per group). Data are presented as the mean ± SD. ***p* < 0.01. *p* values were calculated by the Student’s *t* test. **(G)** Fluorescence intensity of immunostained *K. pneumoniae* per pixel were measured by the ImageJ analysis software. Each dot represents the value from an individual mouse (n = 6 per group). Data are presented as the mean ± SD. ***p* < 0.01. *p* values were calculated by the Student’s *t* test.

**Fig. 2: (A)** An *in vitro K. pneumoniae* infection model using a Transwell insert co-culture system consisting of Caco-2 cells grown on the insert and RAW264.7 macrophages or BMDMs grown in the cell culture well. **(B and C)** Bacterial counts in Caco-2 cells in the presence or absence of RAW264.7 macrophages (B) or BMDMs (C). Caco2 cells were lysed with PBS/1% Triton X-100, and the lysate was plated on LB agar. The number of CFU was counted after 24 h incubation. Each dot represents six independent replicates (n = 6 per group). Data are presented as the mean ± SD. ***p* < 0.01. *p* values were calculated by the Student’s *t* test. **(D)** Caco-2 cells grown on the insert in the presence or absence of RAW264.7 macrophages were immunostained with an anti-E-cadherin antibody and an anti-*Klebsiella pneumoniae* antibody. Each image has five independent replicates. Scale bar = 50 μm. **(E and G)** Caco-2 cells grown on the insert in the presence or absence of RAW264.7 macrophages were immunostained with an anti-E-cadherin antibody, and an anti-ZO-1 antibody (E) or an anti-occludin antibody (G). Each image has five independent replicates. Scale bar = 50 μm. **(F and H)** Fluorescence intensity per pixel was measured by ImageJ analysis software. Each dot represents five independent replicates (n = 5 per group). Data are presented as the mean ± SD. ***p* < 0.01. *p* values were calculated by the Student’s *t* test.

**Fig. 3: (A)** Cytokine array analysis of the *K. pneumoniae* infection model based on the Transwell insert co-culture system consisting of Caco-2 cells and RAW264.7 macrophages. Quantification of Axl signals using a laser scanner. Each dot represents four independent replicates (n = 4 per group). Data are presented as the mean ± SD. ***p* < 0.01. *p* values were calculated by the Student’s *t* test. **(B and C)** Secretion of Axl (B) and Gas6 (C) by Caco-2 cells or RAW264.7 macrophages. Culture supernatants or lysates of *K. pneumoniae* were added to Caco-2 cells or RAW264.7 macrophages for 12 h. The culture media were then collected for ELISA analysis of Axl or Gas6 levels. Each dot represents six independent replicates (n = 6 per group). Data are presented as the mean ± SD. ***p* < 0.01. *p* values were calculated by one way analysis of variance. **(D)** *K. pneumoniae*-infected Caco-2 cells grown on a Transwell insert in the presence or absence of RAW264.7 macrophages were immunostained with anti-Gas6 and anti-Axl antibodies. Each image has six independent replicates. Scale bar = 50 μm. **(E)** Fluorescence intensity per pixel of anti-Axl antibody (left panel) or an anti-Gas6 antibody (right panel) were measured by the ImageJ analysis software. Each dot represents six independent replicates (n = 6 per group). Data are presented as the mean ± SD. ***p* < 0.01. *p* values were calculated by one way analysis of variance.

**Fig. 4: (A)** Western blotting was performed to detect the expression of Axl, Gas6, ZO-1, and occludin in Caco-2 cells infected with *K. pneumoniae* in the presence of an Axl inhibitor (R428) or an anti-Gas6 antibody. Prior to *K. pneumoniae* infection, cells were treated for 3 h with Axl inhibitor (R428; 20 nM) or 1 μg of anti-Gas6 antibody. Each western blotting image represents three independent replicates. **(B)** Western blotting signal intensity was analyzed by ImageJ software. Each dot represents three independent replicates (n = 3 per group). Data are presented as the mean ± SD. ***p* < 0.01. *p* values were calculated by one way analysis of variance. **(C)** Axl inhibitor (R428; 20 nM) or 1 μg of anti-Gas6 antibody were added to Caco-2 cells grown on the insert in the presence of RAW264.7 macrophages for 3 h prior to *K. pneumoniae* infection. Next, Caco-2 cells were immunostained with an anti-ZO-1 antibody and an anti-occludin antibody. Each image has four (group of Caco-2 cells/RAW264.7 cells stained with an anti-ZO-1 antibody) or five independent replicates. Scale bar = 50 μm. **(D)** Fluorescence intensity per pixel of anti-ZO-1 antibody (left panel) or an anti-occludin antibody (right panel) were measured by the ImageJ analysis software. Each dot represents four or five independent replicates (n = 4 or 5 per group). Data are presented as the mean ± SD. ***p* < 0.01. *p* values were calculated by one way analysis of variance. **(E)** Axl inhibitor (R428; 20 nM) or 1 μg of anti-Gas6 antibody were added to Caco-2 cells grown on the insert in the presence or absence of RAW264.7 macrophages for 3 h prior to *K. pneumoniae* infection. Next, Caco-2 cells were immunostained with an anti-*Klebsiella pneumoniae* antibody. Each image has six independent replicates. Scale bar = 50 μm. **(F)** Fluorescence intensity per pixel of *K. pneumoniae* were measured by the ImageJ analysis software. Each dot represents six independent replicates (n = 6 per group). Data are presented as the mean ± SD. ***p* < 0.01. *p* values were calculated by one way analysis of variance. **(G)** Bacterial counts within Caco-2 cells treated with an Axl inhibitor (R428) or an anti-Gas6 antibody in the presence of BMDMs. Prior to *K. pneumoniae* infection, Caco-2 cells were treated for 3 h with Axl inhibitor (R428; 20 nM) or 1 μg of anti-Gas6 antibody. Cells were lysed with PBS/1% Triton X-100, and the lysate was plated on LB agar. The number of CFU was counted after 24 h incubation. Each dot represents six independent replicates (n = 6 per group). Data are represented as mean ± SD. ***p* < 0.01. *p* values were calculated by one way analysis of variance.

**Fig. 5:** **(A and B)** Sections of cecal mucosa (A) or liver (B) were from mice aged 15 or 57 weeks at 2 days after infection with *K. pneumoniae* ATCC43816 pmCherry and immunostained with an anti-Gas6 antibody and an anti-Axl antibody. Each image has six independent replicates. Scale bar = 50 μm. **(C)** Detection of Axl, Gas6, ZO-1, and occludin by western blotting. Cecum and liver tissues were collected, homogenized, and analyzed using an anti-Axl antibody, an anti-Gas6 antibody, an anti-ZO-1 antibody, or an anti-occludin antibody. Each lane of western blotting images represents the protein from an individual mouse (mouse number = 5 (cecum) or 4 (liver) per group). Western blotting image represents three independent replicates. **(D)** Western blotting signal intensities were analyzed by ImageJ software. Each dot represents the value collected from an individual mouse (mouse number = 5 (cecum) or 4 (liver) per group). Data are presented as the mean ± SD. NS: not significant, **p* < 0.05, ***p* < 0.01. *p* values were calculated by the Student’s *t* test. **(E)** Linear correlation between Gas6 expression and Axl expression in the cecum or liver of mice aged 15 (red circles) or 57 (blue circles) weeks infected with *K. pneumoniae* ATCC43816 pmCherry. Each dot represents an ELISA measured value of each protein sample collected from an individual mouse (mouse number = 5 per group). *r* > 0.70 and *p* < 0.05. **(F)** The population of CD11b+F4/80+ macrophages in the intestinal mucosa of young (14-week-old) and old (56-week-old) mice was examined by staining with anti-F4/80 and anti-CD11b antibodies. The percentage of F4/80-positive/CX3CR1-negative cells in 14-week-old and 56-week-old mice was 29.5% and 8.87%, respectively. Data are representative of four mice per group.

**Fig. 6: (A)** Administration of Gas6 recombinant protein to Caco-2 cells to analyze the expression of Axl, ZO-1, and occludin. Addition of Gas6 recombinant protein to the apical surface (left scheme) or the basolateral side (right scheme) of Caco-2 cells. **(B)** Western blot analysis to detect Axl, Gas6, ZO-1, and occludin in Caco-2 cells treated with human Gas6 recombinant protein from the apical or basolateral sides. Each western blotting image represents three independent replicates (n = 3). **(C)** Western blot analysis was performed to detect Axl, Gas6, ZO-1, and occludin. Gas6 recombinant protein (1 μg) was added to the apical side of Caco-2 cells grown in a Transwell co-culture system for 3 h prior to *K. pneumoniae* infection. Each western blotting image represents three independent replicates (n = 3). **(D)** Gas6 recombinant protein (1 μg) was added to Caco-2 cells grown on the Transwell insert for 3 h prior to *K. pneumoniae* infection. The cells were immunostained with an anti-ZO-1 antibody or an anti-occludin antibody, and an anti-E-cadherin antibody. Each image has four independent replicates. Scale bar = 20 μm. **(E)** Fluorescence intensity per pixel of anti-ZO-1 antibody, an anti-occludin antibody, and anti-E-cadherin antibody were measured by ImageJ analysis software. Each dot represents four independent replicates (n = 4 or 5 per group). Data are presented as the mean ± SD. NS: not significant, ***p* < 0.01. *p* values were calculated by the Student’s *t* test. **(F)** Gas6 recombinant protein (1 μg) was added to Caco-2 cells grown on the Transwell insert for 3 h prior to *K. pneumoniae* infection. The cells were immunostained with an anti-*Klebsiella pneumoniae* antibody and an anti-E-cadherin antibody. Each image has four independent replicates. Scale bar = 20 μm. **(G)** Fluorescence intensity per pixel of anti-*Klebsiella pneumoniae* antibody were measured by the ImageJ analysis software. Each dot represents five independent replicates (n = 5 per group). Data are presented as the mean ± SD. ***p* < 0.01. *p* values were calculated by the Student’s *t* test.

**Fig. 7: (A)** Treatment scheme used to analyze the effect of Gas6 recombinant protein on the susceptibility of 57-week-old mice to infection by *K. pneumoniae*. Antibiotics were administered 4 weeks before administration of Gas6 recombinant protein. Gas6 recombinant protein (125 μg protein/kg) was administered intraperitoneally to 57-week-old mice three times every 24 h prior to bacterial infection. Survival was monitored daily (Experiment A). At 2 days post-infection, the mice were sacrificed, and the cecum and liver were harvested (Experiment B). **(B)** Effect of Gas6 recombinant protein on survival of 57-week-old mice infected with *K. pneumoniae* ATCC43816 pmCherry. Each mouse was orally inoculated with *K. pneumoniae* ATCC43816 pmCherry (5 × 10^7^ bacteria). Each dot was represented from an individual mouse (n = 6 per group). *p* values were determined using the log-rank test. **(C)** Bacterial counts in cecal mucosa, liver, and cecal contents were determined 2 days post-infection. Each tissue and cecal contents were homogenized in PBS. The homogenates were plated on LB agar containing 400 μg/mL ampicillin and the number of CFU was counted. Each dot represents the value from an individual mouse (n = 5 or 6 per group). Data are presented as the mean ± SD. **p* < 0.05, ***p* < 0.01. *p* values were calculated by one way analysis of variance. **(D)** Detection of F4/80 by western blotting. Cecal mucosa were collected, homogenized, and analyzed by western blotting with anti-F4/80 antibodies. Each lane of western blotting images represents the protein from an individual mouse (mouse number = 5 per group). Western blotting image represents three independent replicates. **(E)** Western blotting signal intensities were analyzed by ImageJ software. Each dot represents the value collected from an individual mouse (mouse number = 5 per group). Data are presented as the mean ± SD. ** *p* < 0.01. *p* values were calculated by one way analysis of variance. **(F)** Sections of cecal mucosa were from mice aged 15-week, 57-week, or Gas6 administered 57-week-old mice at 2 days after infection with *K. pneumoniae* ATCC43816 pmCherry and immunostained with an anti-Gas6 antibody and an anti-Axl antibody. Each image has six independent replicates. Scale bar = 50 μm. **(G)** Fluorescence intensity was analyzed by ImageJ software. Each dot represents the value from an individual mouse (n = 6 per group). Data are presented as the mean ± SD. ***p* < 0.01. *p* values were calculated by one way analysis of variance. **(H)** Detection of Axl, Gas6, ZO-1, and occludin by western blotting. Cecal mucosa were collected, homogenized, and analyzed by western blotting with anti-Axl, anti-Gas6, anti-ZO-1, or anti-occludin antibodies. Each lane of western blotting images represents the protein from an individual mouse (mouse number = 5 per group). Western blotting image represents three independent replicates. **(I)** Western blotting signal intensities were analyzed by ImageJ software. Each dot represents the value collected from an individual mouse (mouse number = 5 per group). Data are presented as the mean ± SD. NS: not significant, **p* < 0.05, ***p* < 0.01. *p* values were calculated by one way analysis of variance.

**Supporting information**

**Fig S1:** **(A)** H&E staining of cecal mucosa of 15- or 57-week-old mice before initiation of the antibiotic treatment. This image has five independent replicates. Scale bar = 50 μm. **(B)** Mucosal thickness was measured by ImageJ analysis software. Each dot represents the value from an individual mouse (n = 5 per group). Data are presented as the mean ± SD. NS: not significant. **(C)** *K. pneumoniae* ATCC43816 were electroporated with the pmCherry plasmid. This image has seven independent replicates. Scale bar = 5 μm. **(D and F)** H&E staining of cecum (D) and liver (F) tissue from *K. pneumoniae* ATCC43816 pmCherry-infected mice aged 15 or 57 weeks. Desquamation of cecal epithelium is denoted by the black dotted line, and edema of the cecal submucosa is indicated by the red dotted line. Each image has six independent replicates. Scale bar = 50 μm. **(E)** Desquamation and edema length of cecal mucosa was measured by the ImageJ analysis software, and the percentage of desquamation or edema area were calculated. Each dot represents the value from an individual mouse (n = 6 per group). Data are presented as the mean ± SD. **p* < 0.05. *p* values were calculated by the Student’s *t* test. **(G)** Damaged area of liver per total area was measured and percentage calculated by ImageJ analysis software. Each dot represents the value from an individual mouse (n = 6 per group). Data are presented as the mean ± SD. **p* < 0.05. *p* values were calculated by the Student’s *t* test.

**Fig S2:** **(A)** H&E staining of *K. pneumoniae*-infected Caco-2 cells grown on the insert in the presence or absence of RAW264.7 macrophages. The cytoplasmic rupture of Caco-2 cells is indicated by the black dotted line. Each image has six independent replicates. Scale bar = 50 μm. **(B)** Desquamation length of Caco-2 cells grown on the insert was measured by the ImageJ analysis software and the percentage of desquamation area were calculated. Each dot represents six independent replicates (n = 6 per group). Data are presented as the mean ± SD. **p* < 0.05. *p* values were calculated by the Student’s *t* test.

**Fig S3:** **(A and C)** Caco-2 cells grown on the insert in the presence or absence of RAW264.7 macrophages without *K. pneumoniae* infection were immunostained with an anti-E-cadherin antibody, and an anti-ZO-1 antibody (A) or an anti-occludin antibody (C). Each image has five independent replicates. Scale bar = 50 μm. **(B and D)** Fluorescence intensity per pixel of anti-ZO-1 antibody (B) or an anti-occludin antibody (D) were measured by the ImageJ analysis software. Each dot represents five independent replicates (n = 5 per group). Data are presented as the mean ± SD. NS: not significant.

**Fig S4:** **(A)** *K. pneumoniae* infection model using a Transwell insert co-culture system based on Caco-2 cells and BMDMs were constructed. Prior to *K. pneumoniae* infection, Caco-2 cells were treated for 3 h with Axl inhibitor (R428; 20 nM) or 1 μg of anti-Gas6 antibody. Western blotting was performed to detect the expression of Axl, Gas6, ZO-1, and occludin in Caco-2 cells infected with *K. pneumoniae* in the presence of an Axl inhibitor (R428) or an anti-Gas6 antibody. Each western blotting image represents three independent replicates. **(B)** Western blotting signal intensity was analyzed by ImageJ software. Each dot represents three independent replicates (n = 3 per group). Data are presented as the mean ± SD. **p* < 0.05, ***p* < 0.01. *p* values were calculated by one way analysis of variance.

**Fig S5:** **(A)** Axl inhibitor (R428; 20 nM) or 1 μg of anti-Gas6 antibody were added to Caco-2 cells grown on the insert in the presence of RAW264.7 macrophages. Cells were immunostained with an anti-ZO-1 antibody and an anti-occludin antibody. Each image has five independent replicates. Scale bar = 50 μm. **(B)** Fluorescence intensity per pixel of anti-ZO-1 antibody or an anti-occludin antibody were measured by ImageJ analysis software. Each dot represents five independent replicates (n = 5 per group). Data are presented as the mean ± SD. NS: not significant.

**Fig S6:** **(A and B)** Fluorescence intensity per pixel of anti-Gas6 or anti-Axl antibody of cecum (A) and liver (B) were measured by ImageJ analysis software. Each dot represents the value from an individual mouse (n = 6 per group). Data are presented as the mean ± SD. NS: not significant. ** *p* < 0.01. *p* values were calculated by the Student’s *t* test.

**Fig S7:** **(A and B)** Western blotting signal intensity (Fig 6B and 6C) was analyzed by ImageJ software. Each dot represents three independent replicates (n = 3 per group). Data are presented as the mean ± SD. ***p* < 0.01. *p* values were calculated by one way analysis of variance.

**Fig S8:** **(A)** Gas6 recombinant protein (1 μg) was added to Caco-2 cells grown on the Transwell insert for 3 h. Cells were immunostained with an anti-ZO-1 antibody and an anti-occludin antibody. Each image has five independent replicates. Scale bar = 50 μm. **(B)** Fluorescence intensity per pixel of anti-ZO-1 antibody, an anti-occludin antibody, and anti-E-cadherin antibody were measured by the ImageJ analysis software. Each dot represents five independent replicates (n = 5 per group). Data are presented as the mean ± SD. NS: not significant. **p* < 0.05. *p* values were calculated by the Student’s *t* test.

**Fig S9:** **(A)** H&E staining of cecum tissues from *K. pneumoniae* ATCC43816 pmCherry-infected mice. Desquamation of epithelial cells is indicated by the black dotted line. Each image has five independent replicates. Scale bar = 50 μm. **(B)** The area of desquamation was measured by ImageJ software. Each dot represents the value from an individual mouse (n = 5 per group). Data are presented as the mean ± SD. ***p* < 0.01. *p* values were calculated by one way analysis of variance. **(C and D)** Sections of cecal mucosa were from mice aged 15-week, 57-week, or Gas6 administered 57-week-old mice at 2 days after infection with *K. pneumoniae* ATCC43816 pmCherry and immunostained with an anti-F4/80 antibody. Each image has five or six independent replicates. Scale bar = 50 μm. Fluorescence intensity was analyzed by ImageJ software (D). Each dot represents the value from an individual mouse (n = 5 or 6 per group). Data are presented as the mean ± SD. ***p* < 0.01. *p* values were calculated by one way analysis of variance.

**Comment 5.** *Since the authors are activating their RAW264.7 cells with LPS, a no Kp control should be present in all relevant experiments, such as Figs. 2D-E, 4B-C, and 6D-E.*

**(Response)**

Thank you for this comment. In accordance with to your suggestion, we conducted additional immunostaining experiments against *K. pneumoniae*-uninfected samples. As shown in **revised Fig S3**, in *K. pneumoniae*-uninfected Caco-2 cells, ZO-1 and occludin expression was not affected by the presence of RAW264.7 macrophages. Likewise, the expression of ZO-1 and occludin in Caco-2 cells uninfected with *K. pneumoniae* was not affected by the Axl inhibitor or anti-Gas6 antibody (**Revised Fig S5**). Additionally, treatment of Gas6 recombinant protein induced ZO-1 and occludin expression, but not E-cadherin, even in *K. pneumoniae*-uninfected Caco-2 cells (**Revised Fig S8A and S8B**). From these additional results, it was further supported that RAW264.7 macrophages suppress the decrease in ZO-1 and occludin expression in Caco-2 cells by *K. pneumoniae* infection via activating Gas6/Axl signals by Gas6. In Fig 4E and 6F, we did not conduct the immunostaining analysis of *K. pneumoniae* uninfected control samples, because the staining target is *K. pneumoniae* in these figures. These data are shown as Figure S3, S5, and S8 of the revised manuscript, and the Results sections have been revised to include these data. The revised texts that are changed the color within the manuscript are extracted, as follow:

**Line 147**: The ZO-1 and occludin expression in *K. pneumoniae*-uninfected Caco-2 cells was not decreased even in the absence of RAW264.7 macrophages (**S3A, S3B, S3C and S3D Fig**).

**Line 188**: Immunostaining analysis of Caco-2 cells infected with *K. pneumoniae* in the presence of RAW264.7 macrophages revealed a significant decrease in the expression of ZO-1 and occludin by the addition of the Axl inhibitor or anti-Gas6 antibody (**Fig 4C and 4D**). By comparison, in uninfected Caco-2 cells, the expression of ZO-1 and occludin was not affected by the addition of the Axl inhibitor or anti-Gas6 antibody (**S5A and S5B, Fig**).

**Line 240**: Immunostaining analysis revealed that the expression of ZO-1 and occludin (but not E-cadherin) by *K. pneumoniae*-infected Caco-2 cells in the absence of RAW264.7 macrophages increased significantly after treatment with Gas6 recombinant protein (**Fig 6D and 6E**). Also, in uninfected Caco-2 cells, treatment of Gas6 recombinant protein induced ZO-1 and occludin expression, but not E-cadherin (**S8A and S8B Fig**).

**Reviewer #2:**

(No Response)

**Part III – Minor Issues: Editorial and Data Presentation Modifications**

**Reviewer #1**:

**Comment 1.** *There are a few areas where the language can be refined:*

*a. Line 77 - Kp is not classically a gut pathogen. It is a gut colonizer with pathogenic potential. An example of a gut pathogen is C. diff.*

*b. Line 87 - Kp doesn't "target" the elderly. It* *is a more prevalent cause of infection in the elderly.*

**(Response)**

Thank you for the comments. We corrected the phrase as follow;

**Line 77:** The phrase "a gut pathogen" has been corrected to "a gut colonizer with pathogenic potential".

**Line 87:** The word "target" has been revised to "*K. pneumoniae* is a more prevalent cause of infection in the elderly"

**Comment 2.** *Please use log-scale y-axes in Figs. 1C, 2B, 3A, and 7C.*

**(Response)**

Thank you for the comments. We corrected to the log-scale y-axes in these Figures (Figure 1C, 2B, 2C, 3A, 4G, and 7C).

**Comment 3.** *I would encourage the authors to discuss the data shown in Figs. 3B-C. Do the authors think that Kp LPS is the stimulating factor or is it some other factor?*

**(Response)**

Thank you for the comments. We included discussion about the Gas6 inducing factor and revised the Discussion sections. The revised texts that are changed the color within the manuscript are extracted, as follow:

**Line 358**: As shown in Fig 3, *K. pneumoniae* is thought to produce a Gas6-inducing factor. In Caco-2 cells co-cultured with RAW 264.7 macrophages primed by *E. coli*-derived LPS without *K. pneumoniae* infection, Gas6/Axl signaling-dependent enhancement of ZO-1 and occludin expression was not observed (**S3 and S5 Fig**). Thus, the Gas6-inducing factor is thought to be a specific molecule produced by *K. pneumoniae*. Interestingly, since the activity of *K. pneumoniae* to induce Gas6 secretion was not inactivated by heat treatment, it is assumed unlikely that the Gas6-inducing factor is a protein molecule, but rather some specific-LPS or capsule type produced by *K. pneumoniae*. The identification and purification of the Gas6-inducing factor contribute to elucidating the detailed mechanism of Gas6 secretion by macrophages.

**Comment 4.** *Please indicate the oral inoculation method. Oral gavage, feeding, or other?*

**(Response)**

Thank you for the comments. We added the oral inoculation method and revised the Methods sections. The revised texts that are changed the color within the manuscript are extracted, as follow:

**Line 504**: Mice were orally inoculated with 200 μL LB broth containing *K. pneumoniae* ATCC43816 pmCherry (5 × 10^7^ bacteria) using a stainless-steel feeding needle fitted to a 1.0 mL syringe and their survival was monitored daily.

**Comment 5.** *Please indicate the transwell pore size.*

**(Response)**

Thank you for the comments. We added the transwell pore size and revised the Methods sections as follow:

**Line 537**: Caco-2 cells (1 × 10^6^ cells/well) were seeded onto 6-Transwell insert culture plates (0.4 µm pore size) (Corning, Lowell, MA, USA, cat# 3412).

**Comment 6.** *Please indicate if any blinding was performed prior to image analysis and how images were processed and selected for analysis.*

**(Response)**

Thank you for the comments. We added the blinding method, processing, and selection of images for analysis. We revised the Methods sections. The revised text that is changed the color within the manuscript is extracted, as follow:

**Line 472**: Blind analysis of the data was ensured by providing investigators with numbered images. Image data were saved as TIFF files. Images for which the analysis by ImageJ software showed average value results were selected as representative images.

**Reviewer #2:**

**Comment 1.** *Line 105 ff.: Which mouse line was used that carries a commensal K. pneumoniae strain? I am missing some information of the strain used to infect the mice? Which sequence type? Which capsule type? Is the strain hypervirulent, multi drug resistant or both? What are the reasons for choosing this strain? Is this strain relevant in the human population, especially with regard to infection of the elderly?*

**(Response)**

Thank you for the comment. In this study, we used *K. pneumoniae* ATCC43816 strain because it is a well-studied hypervirulent laboratory strain which has K2 serotype capsules and is capable of causing an acute respiratory disease in animal models (***PLoS One***, 9: e107394, 2014). Throughout this study, C57BL/6J male mice, which were purchased from SLC Japan, were used for *in vivo K. pneumoniae* infection models. To distinguish between endogenous (carried a commensal) *K. pneumoniae* and exogenous (orally infected) *K. pneumoniae*, we electroporated *K. pneumoniae* ATCC43816 with the pmCherry plasmid (**S1C Fig**). In an experiment to determine the bacterial number in infected mice, orally administered *K. pneumoniae* ATCC43816 pmCherry was selectively measured by culturing on ampicillin-containing LB plates. Recently, genomic sequencing analysis revealed that *K. pneumoniae* ATCC43816 possess the known virulence determinant for a siderophore-dependent iron uptake system (yersiniabactin), a Type 4 secretion system, and a CRISPR system, which are absent from non-hypervirulent strains (***PLoS One***, 9: e107394, 2014). *K. pneumoniae* ATCC43816 is a hypervirulent strain, and therefore garners attention due to its ability to infect younger, healthy individuals. In this study, we show that Gas6 inhibits the invasion of *K. pneumoniae* ATCC43816 to the intestinal mucosa by enhancing tight-junction barriers in the intestinal epithelium, contributing to the prevention of bacterial translocation. Specifically, the activity of Gas6 prevents the bacterial translocation of hypervirulent strains, and therefore the present results would be a beneficial for preventing *K. pneumoniae* infection. On the other hand, further studies are needed to determine whether Gas6 also prevents the bacterial translocation of non-hypervirulent *K. pneumoniae* strains from the gastrointestinal tract, which are particularly prevalent in the elderly. The Results and Discussion sections have been revised to include this information. The revised texts that are changed the color within the manuscript are extracted, as follow:

**Line 108**: To distinguish between endogenous (a gut commensal) *K. pneumoniae* and exogenous (orally introduced) *K. pneumoniae*, we electroporated *K. pneumoniae* ATCC43816 with the pmCherry plasmid (**S1C Fig**) and then infected the mice.

**Line 116**: In an experiment to determine the bacterial number in infected mice, orally infected *K. pneumoniae* ATCC43816 pmCherry was selectively measured by culturing on ampicillin-containing LB plates.

**Line 290**: *K. pneumoniae* ATCC43816 is a well-studied hypervirulent laboratory strain which has K2 serotype capsules and can cause an acute respiratory disease in animal models (***PLoS One***, 9: e107394, 2014). Recently, genomic sequencing analysis revealed that *K. pneumoniae* ATCC43816 possess the known virulence determinant for a siderophore-dependent iron uptake system (yersiniabactin), a Type 4 secretion system, and a CRISPR system, which are absent from non-hypervirulent strains (***PLoS One***, 9: e107394, 2014). In this study, we show that Gas6 inhibits the invasion of *K. pneumoniae* ATCC43816 to the intestinal epithelium by enhancing tight-junction barriers, contributing to the prevention of the bacterial translocation. Since hypervirulent strains have the ability to infect young, healthy individuals, the activity of Gas6 in preventing the bacterial translocation of hypervirulent strains is beneficial information to prevent *K. pneumoniae* infection.

*K. pneumoniae* is reported to invade human epithelial cell lines. The invasion rates among each clinically isolated strain range from 10.1 – 48.6%, indicating that invasive abilities are differ among the bacterial strains (***Infection and immunity***, 65:4445–4451, 1997, ***Infection and immunity***, 65:2950-2958, 1997). The difference in invasiveness is reported to not be dependent on capsule type, suggesting that invasion abilities are mediated by multifactorial process and that capsule alone is insufficient to account for differences in pathogenicity between strains (***Infection and immunity***, 65:4445–4451, 1997). Pan *et al*. also reported that additional genetic loci, including genes for lipopolysaccharides (LPS), the ClpX protease, and carnitine metabolism, contribute to the virulence of *K. pneumoniae* (***Infection and immunity***, 79:997-1006, 2011). From these observations, it was thought that virulence factors of *K. pneumoniae* are diverse and it may be difficult to identify the factors which determined the difference in virulence among the bacterial strains. The protective ability of Gas6 to the invasion of *K. pneumoniae* depends on the Gas6/Axl signaling in epithelial cells, and therefore Gas6 can be inferred to prevent the bacterial invasion regardless of the bacterial strain. Further studies are needed to determine whether Gas6 also prevents the bacterial translocation from the gastrointestinal tract against non-hypervirulent *K. pneumoniae* strains, which are particularly prevalent in the elderly, by analyzing the inducibility of Gas6 secretion from macrophages by several strains.

**Comment 2.** *Line 118ff.: If the phenotype is really exclusively driven by the number of intestinal macrophages, the authors should exclude other potential influencing factors like age dependent differences in the microbiota, the mucosal architecture and other immune-related factors that has been described for K. pneumoniae-host immune system-interplay (Sequeira et al., 2020 DOI: 10.1038/s41564-019-0640-1, Constantinides et al., 2019 DOI: 10.1126/science.aax6624). I would suggest to generate a more holistic data set of the used animals. How does the microbiome composition of the mice look before initiation of antibiotic treatment? Did the microbiome composition also change within the lifespan of the animals? More specifically, is the alpha diversity already reduced in elderly mice? If I understand correct, mice receive a heavy antibiotics cocktail over the whole course of experiment, which in turn would lead to a massive reduction of the present microbes in the gut and might affect the abundance and composition of relevant immune cell subsets. Which other bacteria, except of the commensal K. pneumoniae strain are actually still present? Is the status of being germfree validated? If the purpose is to completely eradicate the gut microbes, why not use germfree animals instead? Why did the author chose a mouse model harboring an commensal K. pneumoniae strain in the gut? Is the immune system not already primed for K. pneumoniae then?*

**(Response)**

Thank you for the comment. Our results showed that Gas6 secreted by macrophage inhibits the invasion of *K. pneumoniae* into the intestinal epithelium by increasing expression of tight-junction proteins. Gas6 was hardly detected in the intestinal mucosa of elderly mice under *K. pneumoniae* infection, and then the bacterial translocation from gastrointestinal tract to the liver was induced. Additionally, administration of Gas6 recombinant protein to the elderly mice significantly prevented the bacterial translocation from the gastrointestinal tract. From these findings, we concluded that Gas6 secreted from macrophages plays a pivotal role in the protection against *K. pneumoniae* infection in elderly. However, we do not believe that enhanced susceptibility to *K. pneumoniae* with aging is determined solely by a decrease in the Gas6-producing macrophages. As this comment by Reviewer #2 indicated, there are experimental limitations in our *in vivo K. pneumoniae* infection models. Host defense mechanisms are maintained by a variety immune-related factors, and these immune-factors are influenced and regulated by the gut microbiota (***Science***, 336:1268-1273, 2012, DOI: 10.1126/science.1223490). The influence of gut microbiota to the host not only fosters development and aids digestion but also directly protects the host from infection by pathogens, which is a function referred to as colonization resistance (***Trends in Microbiology***, 16:107-114, 2008, DOI: 10.1016/j.tim.2007.12.008). We recently reported that microbiota-derived short-chain fatty acids (SCFAs; propionate and butyrate) promoted inflammasome activation in macrophages by binding to its apoptosis-associated speck-like protein (ASC), leading to the enhanced elimination of *Salmonella* *enterica* serovar Typhimurium (*S. Typhimurium*) within macrophages (***PLoS Biol.***, 18: e3000813, 2020, DOI: 10.1371/journal.pbio.3000813). Dr. Sequeira *et al*. reported that *Bacteroidetes* specifically protects against intestinal colonization by *K. pneumoniae* through IL-36 signaling (***Nature Microbiology***, 5: 304–313, 2020, DOI: 10.1038/s41564-019-0640-1). In this study, to exclude the effects of colonization resistance by microbiota on the *K. pneumoniae* infection, antibiotic-treated mice were used. Rakoff-Nahoum and colleagues reported that the combination therapy of four antibiotics (ampicillin, metronidazole, neomycin, and vancomycin) for mice was used for complete depletion of gut commensal bacteria (***Cell***, 118:229-41, 2004, DOI: 10.1016/j.cell.2004.07.002.). Therefore, in this study, each mouse was given drinking water containing ampicillin (1 g/L), metronidazole (1 g/L), neomycin (1 g/L), and vancomycin (0.5 g/L) for 4 weeks prior to *K. pneumoniae* infection (***Immunity***, 29:637-49, 2008, DOI: 10.1016/j.immuni.2008.08.009.). Our previous study confirmed that this antibiotic treatment significantly decreases the concentrations of gut microbiota-derived SCFAs (propionate and butyrate) in the cecal lumen (***PLoS Biol.***, 18: e3000813, 2020, DOI: 10.1371/journal.pbio.3000813). Additionally, in our other study, the microbiome composition of mice before and after antibiotic treatment has been shown to differ (*Figure below*). As shown in the below Figure, antibiotics treatment induces a drastic alteration of gut microbiota including depletion of *Bacteroidetes*. Using in this mouse model, Gas6 expression was enhanced in the cecal mucosa of 15-week-old mice infected with *K. pneumoniae*, suggesting that Gas6 expression under *K. pneumoniae* infection is the event independently affecting the composition of gut microbiota (**Fig 5A, 5C and 5D**). Although we need to confirm whether the protective effect of Gas6 is affected by changes in the gut microbiota with aging using the elderly specific-pathogen-free (SPF)-mice, because the composition of the gut microbiota is known to change gradually with age (***Science***, 350:1214-5, 2015, DOI: 10.1126/science.aac8469, ***PNAS***,108:4586-91, 2011, DOI: 10.1073/pnas.1000097107), our results reveal a novel host-defense mechanisms in which Gas6 secreted by macrophages protects against *K. pneumoniae* infection, in addition to the defensive mechanisms by *Bacteroidetes*. Further studies using germ-free mice would be useful to exclude the four other potential factors which could influenced the protective activity of Gas6: (1) Effect of changing the abundance and composition of relevant immune cell subsets, such as reduced secretion of antimicrobial peptides and changes in T helper cell populations, by antibiotic administration (***Nature Reviews Microbiology***, 9:233–243, 2011, DOI:10.1038/nrmicro2536.), (2) Effect of the difference in composition of the immune system influenced by early encounters with microorganisms (***Science***, 366: 6464, 2019, DOI: 10.1126/science.aax6624.), (3) Effect of the immune-systems modulated by early priming for *K. pneumoniae*, (4) The possibility that gut microbiota in mice treated with antibiotics were not completely eradicated. From the study using germ-free mice, it is expected that detailed information on the benefit of Gas6 for the protective and/or therapeutic activity against *K. pneumoniae* infection in the elderly would be revealed.

This information is addressed in the Discussion sections, and the Discussion sections have been revised to include this information. The revised texts that are changed the color within the manuscript are extracted, as follows:

**Line 315:** Host defense mechanisms are maintained by a variety immune-related factors influenced and regulated by the gut microbiota (***Science***, 336:1268-1273, 2012, DOI: 10.1126/science.1223490). The influence of gut microbiota to the host not only fosters development and aids digestion but also directly protects the host from infection by pathogens, a function referred to colonization resistance to pathogens (***Trends in Microbiology***, 16:107-114, 2008, DOI: 10.1016/j.tim.2007.12.008). We recently reported that short-chain fatty acids (SCFAs), which are metabolites of gut microbiota, promoted inflammasome activation in macrophages by binding to its apoptosis-associated speck-like protein (ASC), leading to the enhanced elimination of *Salmonella* *enterica* serovar Typhimurium (*S. Typhimurium*) within macrophages (***PLoS Biol.***, 18: e3000813, 2020, DOI: 10.1371/journal.pbio.3000813). Dr. Sequeira *et al*. reported that *Bacteroidetes* specifically protects against intestinal colonization by *K. pneumoniae* through IL-36 signaling (***Nature Microbiology***, 5: 304–313, 2020, DOI: 10.1038/s41564-019-0640-1). In this study, to exclude the effects of colonization resistance by microbiota on the *K. pneumoniae*, antibiotic-treated mice were used. Rakoff-Nahoum and colleagues reported that the combination therapy of four antibiotics (ampicillin, metronidazole, neomycin, and vancomycin) for mice was used for complete depletion of gut commensal bacteria (***Cell***, 118:229-41, 2004, doi: 10.1016/j.cell.2004.07.002.). This combination of antibiotics significantly decreases the concentrations of SCFAs (propionate and butyrate) in the cecal lumen (***PLoS Biol.***, 18: e3000813, 2020, DOI: 10.1371/journal.pbio.3000813). Using mice treated with a combination of four antibiotics, induction of Gas6 expression was detected in the cecal mucosa of 15-week-old mice infected with *K. pneumoniae*, suggesting that Gas6 expression, under *K. pneumoniae* infection, is the event that independently affects the composition of gut microbiota (**Fig 5A, 5C and 5D**). Our results reveal a novel host-defense mechanisms in which Gas6 secreted by macrophages protects against *K. pneumoniae* infection, in addition to the defensive mechanisms by *Bacteroidetes*.

Further studies using germ-free mice would be useful to exclude the four other potential factors which could influence the protective activity of Gas6: (1) Effect of changing the abundance and composition of relevant immune cell subsets, such as reduced secretion of antimicrobial peptides and changes in T helper cell populations, by administration of antibiotics (***Nature Reviews Microbiology***, 9:233–243, 2011, DOI:10.1038/nrmicro2536.), (2) Effect of the difference in composition of the immune system influenced by early encounters with microorganisms (***Science***, 366: 6464, 2019, DOI: 10.1126/science.aax6624.), (3) Effect of the immune-systems modulated by early priming for *K. pneumoniae*, (4) The possibility that gut microbiota in antibiotic-treated mice were not completely eradicated. From the study using germ-free mice, it is expected that detailed information on the benefit of Gas6 for the protective and/or therapeutic activity against *K. pneumoniae* infection in the elderly would be revealed.

**Comment 3.** *Figure 1+7: How many mice have been used? It looks like a single experiment has been performed with only 5 mice, which is in my point of view a very limited samples set. It would be worth repeating it at least once. It would be also interesting to see how different CFU counts of K. pneumoniae are in the lumen. If the number of microbial cells is already stronger reduced in younger mice, it would also mean that the general colonization resistance is general higher in younger mice meaning tissue infiltration is also less pronounced. Also, in Figure 1, if mice were sacrificed on day 2 after infection, how could the authors monitor the survival over 16 days? (I don’t understand the cartoon in A and the resulting graph in B). Have the authors checked the initial status of the mucosal architecture in the mice of different age before initiation of the antibiotics course? If the mucosal thickness is already different it would be also another explanation for the observed differences regarding tissue infiltration.*

**(Response)**

Thank you for the comment. In animal experiments, two independent experiments were performed on different days. First experiment (5 or 6 mice per group) was performed for collecting preliminary data and second experiment (5 or 6 mice per group) was performed for data collection. We confirmed that similar results were obtained in two independent animal experiments. We conducted additional experiments to examine whether the infected-bacterial number in the cecal lumen was affected by aging or administration of Gas6 recombinant protein (**Fig 7C**). Results, showed no difference in the infected-bacterial number in the cecal contents between 15-week- and 57-week-old mice (**Fig 7C**). Additionally, the infected-bacterial number in the cecal contents was not affected by the administration of Gas6 recombinant protein, suggesting that aging and administration of Gas6 does not directly reduce the bacterial load (**Fig 7C**). In Figure 1 and 7, the experiments for monitoring the survival (Experiment A) and the experiments for harvesting tissues at 2 days post-infection (Experiment B) were performed separately on different days. We revised the cartoon in Fig 1A and 7A. We conducted additional experiments to check the initial status of the mucosal architecture in the 15-week-, and 57-weekold mice. As shown in revised **Figure S1A and S1B**, there was no detected a difference of the cecal mucosal architecture and thickness between 15-week-, and 57-week-old mice before initiation of the antibiotic treatments. These data are shown as Figure S1 of the revised manuscript, and the Results, Methods and Figure Legends sections have been revised to include these data. The revised texts that are changed the color within the manuscript are extracted, as follow:

**Line 106**: As shown in Fig S1A and S1B, no difference of the cecal mucosal architecture and thickness detected between 15-week-, and 57-week-old mice before initiation of the antibiotic treatment.

**Line 259**: There was no difference of the bacterial number in the cecal contents between 15-week- and 57-week-old mice, suggesting that the aging does not affect the colonization of the bacteria in antibiotics-administered mice (**Fig 7C**).

**Line 468**: In animal experiments, two independent experiments were performed on different days. The first experiment was performed to collect preliminary data and the second experiment was performed for data collection. The number of representative image replicates, and the number of each sample, is described within the relevant Figure legend.

**Legends of Fig 1: (A)** Treatment scheme used to analyze age-related differences in susceptibility to *K. pneumoniae*. Mice aged 15- or 57-week were administered antibiotics for 4 weeks prior to oral inoculation of *K*. *pneumoniae* ATCC43816 pmCherry (5 × 10^7^ bacteria). Survival was monitored daily (Experiment A). At 2 days post-infection, the mice were sacrificed, and the cecum and liver were harvested (Experiment B). **(B)** Effect of aging on the survival of mice infected with *K. pneumoniae* ATCC43816 pmCherry. Each dot was represented from an individual mouse (n = 6 per group). *p*-values were determined using the log-rank test. **(C)** Bacterial counts in the cecum and liver were determined 2 days after infection. Cecum and liver tissues were homogenized in PBS, and the homogenates were plated onto LB agar containing 400 μg/mL ampicillin. The number of CFU was determined. Each dot represents the value from an individual mouse (n = 5 per group). Data are presented as the mean ± SD. **p* < 0.05, ***p* < 0.01. *p* values were calculated by Student’s *t* test. **(D and F)** Sections of cecal mucosa (D) or liver (F) were from mice aged 15 or 57 weeks at 2 days after infection with *K. pneumoniae* ATCC43816 pmCherry and immunostained with an anti-mCherry antibody and rhodamine phalloidin staining. Each image has six independent replicates. Scale bar = 20 μm. **(E)** Number of immunostained puncta of *K. pneumoniae* within the cecal submucosa per same area were counted by the ImageJ analysis software. Each dot represents the value from an individual mouse (n = 6 per group). Data are presented as the mean ± SD. ***p* < 0.01. *p* values were calculated by the Student’s *t* test. **(G)** Fluorescence intensity of immunostained *K. pneumoniae* per pixel were measured by the ImageJ analysis software. Each dot represents the value from an individual mouse (n = 6 per group). Data are presented as the mean ± SD. ***p* < 0.01. *p* values were calculated by the Student’s *t* test.

**Legends of Fig 5: (A and B)** Sections of cecal mucosa (A) or liver (B) were from mice aged 15 or 57 weeks at 2 days after infection with *K. pneumoniae* ATCC43816 pmCherry and immunostained with an anti-Gas6 antibody and an anti-Axl antibody. Each image has six independent replicates. Scale bar = 50 μm. **(C)** Detection of Axl, Gas6, ZO-1, and occludin by western blotting. Cecum and liver tissues were collected, homogenized, and analyzed using an anti-Axl antibody, an anti-Gas6 antibody, an anti-ZO-1 antibody, or an anti-occludin antibody. Each lane of western blotting images represents the protein from an individual mouse (mouse number = 5 (cecum) or 4 (liver) per group). Western blotting image represents three independent replicates. **(D)** Western blotting signal intensities were analyzed by ImageJ software. Each dot represents the value collected from an individual mouse (mouse number = 5 (cecum) or 4 (liver) per group). Data are presented as the mean ± SD. NS: not significant, **p* < 0.05, ***p* < 0.01. *p* values were calculated by the Student’s *t* test. **(E)** Linear correlation between Gas6 expression and Axl expression in the cecum or liver of mice aged 15 (red circles) or 57 (blue circles) weeks infected with *K. pneumoniae* ATCC43816 pmCherry. Each dot represents an ELISA measured value of each protein sample collected from an individual mouse (mouse number = 5 per group). *r* > 0.70 and *p* < 0.05. **(F)** The population of CD11b+F4/80+ macrophages in the intestinal mucosa of young (14-week-old) and old (56-week-old) mice was examined by staining with anti-F4/80 and anti-CD11b antibodies. The percentage of F4/80-positive/CX3CR1-negative cells in 14-week-old and 56-week-old mice was 29.5% and 8.87%, respectively. Data are representative of four mice per group.

**Legends of Fig 7:** **(A)** Treatment scheme used to analyze the effect of Gas6 recombinant protein on the susceptibility of 57-week-old mice to infection by *K. pneumoniae*. Antibiotics were administered 4 weeks before administration of Gas6 recombinant protein. Gas6 recombinant protein (125 μg protein/kg) was administered intraperitoneally to 57-week-old mice three times every 24 h prior to bacterial infection. Survival was monitored daily (Experiment A). At 2 days post-infection, the mice were sacrificed, and the cecum and liver were harvested (Experiment B). **(B)** Effect of Gas6 recombinant protein on survival of 57-week-old mice infected with *K. pneumoniae* ATCC43816 pmCherry. Each mouse was orally inoculated with *K. pneumoniae* ATCC43816 pmCherry (5 × 10^7^ bacteria). Each dot was represented from an individual mouse (n = 6 per group). *p* values were determined using the log-rank test. **(C)** Bacterial counts in cecal mucosa, liver, and cecal contents were determined 2 days post-infection. Each tissue and cecal contents were homogenized in PBS. The homogenates were plated on LB agar containing 400 μg/mL ampicillin and the number of CFU was counted. Each dot represents the value from an individual mouse (n = 5 or 6 per group). Data are presented as the mean ± SD. **p* < 0.05, ***p* < 0.01. *p* values were calculated by one way analysis of variance. **(D)** Detection of F4/80 by western blotting. Cecal mucosa were collected, homogenized, and analyzed by western blotting with anti-F4/80 antibodies. Each lane of western blotting images represents the protein from an individual mouse (mouse number = 5 per group). Western blotting image represents three independent replicates. **(E)** Western blotting signal intensities were analyzed by ImageJ software. Each dot represents the value collected from an individual mouse (mouse number = 5 per group). Data are presented as the mean ± SD. ** *p* < 0.01. *p* values were calculated by one way analysis of variance. **(F)** Sections of cecal mucosa were from mice aged 15-week, 57-week, or Gas6 administered 57-week-old mice at 2 days after infection with *K. pneumoniae* ATCC43816 pmCherry and immunostained with an anti-Gas6 antibody and an anti-Axl antibody. Each image has six independent replicates. Scale bar = 50 μm. **(G)** Fluorescence intensity was analyzed by ImageJ software. Each dot represents the value from an individual mouse (n = 6 per group). Data are presented as the mean ± SD. ***p* < 0.01. *p* values were calculated by one way analysis of variance. **(H)** Detection of Axl, Gas6, ZO-1, and occludin by western blotting. Cecal mucosa were collected, homogenized, and analyzed by western blotting with anti-Axl, anti-Gas6, anti-ZO-1, or anti-occludin antibodies. Each lane of western blotting images represents the protein from an individual mouse (mouse number = 5 per group). Western blotting image represents three independent replicates. **(I)** Western blotting signal intensities were analyzed by ImageJ software. Each dot represents the value collected from an individual mouse (mouse number = 5 per group). Data are presented as the mean ± SD. NS: not significant, **p* < 0.05, ***p* < 0.01. *p* values were calculated by one way analysis of variance.

**Comment 4.** *Line 114ff what about luminal vs tissue burden of K. pneumoniae? The data panels in figure 1 and 7 are relatively empty. I would generate some more data on the differences in the immune system of young and elderly mice.*

**(Response)**

Thank you for the comment. We conducted additional experiments to examine whether the infected-bacterial number in the cecal lumen was affected by aging using antibiotic-treated mice (**Fig 7C**). As shown in Fig 7C, there was no difference in the infected-bacterial load in the cecal contents between 15-week- and 57-week-old mice. The results suggest that in the absence or dysbiosis of gut microbiota, the colonization resistance to orally infected *K. pneumoniae* into the cecal lumen is not affected by aging. There is a lot of evidence that the immune system deteriorates with age, elderly persons are less able to initiate an immune response following pathogenic infections (***Nat Immunol.***, 5:133–139, 2004, ***J. Leukoc. Biol.***, 76: 291-299, 2004, ***Current Opinion Immunol.***, 17: 457-462, 2005). Immunosenescence is known to contribute to not only adaptive immunity dysfunction but also altered innate immunity (***Aging Cell***, 3:195-208, 2004, ***Ageing Res. Rev.***, 2: 419-432, 2003, ***Exp. Gerontol.***, 37: 1325-1331, 2002, ***Aging Cell***, 3: 217-226, 2004). Elucidating the mechanisms of dysfunctional immune systems that contribute to increased susceptibility to *K. pneumoniae* is important and necessary to understand *K. pneumoniae* infection in elderly patients. In this study, we focused on the role of Gas6 secreted from macrophages under *K. pneumoniae* infection. In the future, we will conduct the experiments to analyze the differences in the immune system of young and elderly mice which contribute to the susceptibility to *K. pneumoniae*. Again, thank you very much for the interesting comment. This is addressed in the Discussion section. The revised text that is changed the color within the manuscript is extracted, as follows:

**Line 382**: Several reports show the immune system deteriorates with age, immunosenescence was revealed to contribute to not only adaptive immunity dysfunction but also altered innate immunity (***Nat Immunol.***, 5:133–139, 2004, ***Current Opinion Immunol.***, 17: 457-462, 2005). Our results show that in the antibiotic-treated mice, the colonization resistance to orally infected *K. pneumoniae* into the cecal lumen is not affected by aging (**Fig 7C**). Elucidating the dysfunctional mechanisms of immune systems that contribute to increased susceptibility to *K. pneumoniae* is important and necessary to understand *K. pneumoniae* infection in the elderly patients. Although the present study shows the protective effects of Gas6 secreted from macrophages against *K. pneumoniae* infection in elderly mice, further studies are needed to determine the differences in the immune system of young and elderly mice which contribute to the susceptibility to *K. pneumoniae*.

**Comment 5.** *How broadly applicable is this phenotype? Klebsiella pneumoniae strains are highly diverse (also stated by the authors). Does the exogenous application of Gas6 also prevents tissue infiltration of other hypervirulent K. pneumoniae strains? I would suggest to at least repeat the in vitro assay with another relevant human strain of K. pneumoniae.*

**(Response)**

Thank you for the important comment. In this study, we show that Gas6 inhibits the invasion of *K. pneumoniae* ATCC43816, hypervirulent strains, to the intestinal mucosa by enhancing tight-junction barriers in the intestinal epithelium, contributing to the prevention of bacterial translocation. Hypervirulent strains have the ability to infect not only elderly individuals but also young, healthy individuals, and therefore we believed that the protective activity of Gas6 in preventing the bacterial translocation of hypervirulent strains is beneficial information for preventing *K. pneumoniae* infection. *K. pneumoniae* is reported to invade human epithelial cell lines and the invasion rates among each clinically isolated strain range from 10.1–48.6%, indicating that invasive abilities differ among bacterial strains (***Infection and immunity*** 65:4445–4451, 1997, ***Infection and immunity*** 65:2950-2958, 1997). The inhibitory effect of Gas6 against invasion of *K. pneumoniae* depends on the Gas6/Axl signals in epithelial cells, and thus Gas6 can be inferred to prevent the bacterial invasion regardless of the bacterial strain. Further studies are needed to determine whether Gas6 also prevents bacterial translocation from the gastrointestinal tract of non-hypervirulent *K. pneumoniae* strains, which are particularly prevalent in the elderly. In the future, we will examine the inducibility of Gas6 secretion from macrophages among different bacterial strains. These important points have been specifically addressed in Discussion sections and revised. The revised texts that are changed the color within the manuscript are extracted, as follow:

**Line 294**: In this study, we show that Gas6 inhibits the invasion of *K. pneumoniae* ATCC43816 to the intestinal mucosa by enhancing tight-junction barriers in the intestinal epithelium, contributing to the prevention of the bacterial translocation. Since hypervirulent strains have the ability to infect young, healthy individuals, the activity of Gas6 in preventing the bacterial translocation of hypervirulent strains is beneficial information to prevent *K. pneumoniae* infection.

*K. pneumoniae* is reported to invade human epithelial cell lines. The invasion rates among each clinically isolated strain range from 10.1 – 48.6%, indicating that invasive abilities are differ among the bacterial strains (***Infection and immunity***, 65:4445–4451, 1997, ***Infection and immunity***, 65:2950-2958, 1997). The difference in invasiveness is reported to not be dependent on capsule type, suggesting that invasion abilities are mediated by multifactorial process and that capsule alone is insufficient to account for differences in pathogenicity between strains (***Infection and immunity***, 65:4445–4451, 1997). Pan *et al*. also reported that additional genetic loci, including genes for lipopolysaccharides (LPS), the ClpX protease, and carnitine metabolism, contribute to the virulence of *K. pneumoniae* (***Infection and immunity***, 79:997-1006, 2011). From these observations, it was thought that virulence factors of *K. pneumoniae* are diverse and it may be difficult to identify the factors which determined the difference in virulence among the bacterial strains. The protective ability of Gas6 to the invasion of *K. pneumoniae* depends on the Gas6/Axl signaling in epithelial cells, and therefore Gas6 can be inferred to prevent the bacterial invasion regardless of the bacterial strain. Further studies are needed to determine whether Gas6 also prevents the bacterial translocation from the gastrointestinal tract against non-hypervirulent *K. pneumoniae* strains, which are particularly prevalent in the elderly, by analyzing the inducibility of Gas6 secretion from macrophages by several strains.

**Discussion**

**Comment 6.** *Line 250 the word symbiont implicates that the bacteria are beneficial for the host, better use pathobiont.*

**(Response)**

Thank you for the comments. The word "symbiont" has been corrected to "pathobiont".

**Comment 7*.*** *Line 284 the authors claim potential use of exogenous Gas6 to treat infection in the elderly community. But all the mouse models used by the authors rely on heavy antibiotics treatment, which in my point of view does not reflect the homeostatic conditions we find in the healthy community. Is this model also working in antibiotic naïve mice?*

**(Response)**

Thank you for the comments. As you have mentioned, administration of antibiotics is reported to change the abundance and composition of immune cell subsets (***Nature Reviews Microbiology***, 9:233–243, 2011, DOI:10.1038/nrmicro2536.). Therefore, we will need to examine the protective ability of exogenous Gas6 to specific-pathogen-free (SPF)-mice infected with *K. pneumoniae* in the future. This is addressed in the Discussion section. The revised texts that are changed the color within the manuscript are extracted, as follow:

**Line 402**: Antibiotic-treated mice were used for the *in vivo* infection model in this study. Administration of antibiotics is reported to change the abundance and composition of immune cell subsets (***Nature Reviews Microbiology***, 9:233–243, 2011). Therefore, to determine whether Gas6 has an effect, even in a healthy homeostatic environment in the gut, we also need to examine the protective ability of exogenous Gas6 to SPF-mice infected with *K. pneumoniae*.
